# *H-*phosphinic analogs of natural amino acids: a novel and efficient treatment for preventing biodeterioration of treasured painted artworks

**DOI:** 10.3389/fmicb.2026.1677277

**Published:** 2026-04-02

**Authors:** Alexander A. Zhgun, Maxim A. Khomutov, Darya A. Avdanina, Egor Troyan, Maria V. Dumina, Anna A. Ermolyuk, Nikolay Simonenko, Kirill Shumikhin, Elena N. Khurs, Yuliya Zhuikova, Valery P. Varlamov, Mikhail V. Shitov, Alex R. Khomutov

**Affiliations:** 1 Research Center of Biotechnology, Skryabin Institute of Bioengineering, Russian Academy of Sciences, Moscow, Russia; 2 Engelhardt Institute of Molecular Biology, Russian Academy of Sciences, Moscow, Russia; 3 State Tretyakov Gallery, Moscow, Russia; 4 Kurnakov Institute of General and Inorganic Chemistry, Russian Academy of Sciences, Moscow, Russia

**Keywords:** antiseptics, biocides, biodeterioration of cultural heritage, fungi, fungicides, *H*-phosphinic analogs of natural amino acids

## Abstract

**Introduction:**

Microorganisms can destroy various materials that make up objects of cultural heritage. In particular, ancient tempera paintings are made with egg yolk, animal glue, and a number of other organic materials, which serve as a good breeding ground for the development of microorganisms. Recently, the range of traditional antiseptics used to protect tempera paintings from biodeterioration has been significantly reduced because of undesirable properties associated with their interaction with painting materials and toxicity. Therefore, it is necessary to develop a new generation of antiseptics that can effectively protect paintings from destructive microorganisms.

**Methods:**

To solve this challenging task and protect paintings from fungal damage, we used *H*-phosphinic analogs of natural amino acids. Twelve different *H*-phosphinic analogs of natural amino acids were screened on Czapek–Dox agar medium against 11 mold fungi belonging to the genera *Aspergillus*, *Penicillium*, *Simplicillium*, *Microascus*, *Cladosporium*, and *Ulocladium*. These mold fungi are responsible for the biodegradation of tempera paintings and are the dominant representatives of the microbiome of the State Tretyakov Gallery in Russia.

**Results:**

All the studied compounds at concentrations of 0.7–2.5 mM inhibited the mycelial growth of mold fungi. The supplementation of *H*-phosphinic analogs of alanine, aspartate, and valine resulted in the loss of characteristic pigmentation of *Penicillium chrysogenum*, which may be associated with inhibition of Ac-CoA and malonyl-CoA biosynthesis. The *H*-phosphinic analog of methionine protected mock layers with sturgeon glue more effectively than the other *H*-phosphinates and standard antiseptics, such as benzalkonium chloride or sodium pentachlorophenolate. The addition of *H*-phosphinic amino acid analogs to sturgeon glue did not significantly affect the spectral and surface properties of the glue applied on the layout but effectively inhibited the growth of the studied mold fungi on mock-up layers during long-term storage.

**Conclusion:**

Our data provide the first evidence of the successful use of nontoxic *H*-phosphinic analogs of natural amino acids for protecting paintings from biodeterioration.

## Introduction

Molds are a major factor causing biodeterioration of cultural heritage objects, artwork, and historical artifacts (De Leo and Isola, 2022; Zucconi et al., 2022; Szczepanowska, 2023; Gadd et al., 2024). This is because these chemoorganotrophic organisms can use a wide variety of substrates for their development, with various organics as an energy source (Warscheid and Braams, 2000; Branysova et al., 2022; Leplat et al., 2025). During colonization, fungi are capable of causing both physical and chemical damage to cultural heritage objects (Scheerer et al., 2009; Negi and Sarethy, 2019; Zhang et al., 2019; Nitiu et al., 2020). Consequently, sharp-cut regulations have been developed for the conservation and restoration of cultural heritage objects, including the use of a variety of antiseptics that effectively affect molds (Palla and Barresi, 2017; Kakakhel et al., 2019; Avdanina and Zhgun, 2024). However, recently, the palette of antiseptics used to protect artworks from biodeterioration has been significantly reduced (Unger et al., 2001; Sterflinger and Piñar, 2013) because most biocides were not directly developed to protect heritage materials but were borrowed from medicine and agriculture (Franco-Castillo et al., 2021; Kosel et al., 2024). Unfortunately, toxicity and undesirable interactions of a number of these compounds with painting materials, leading to pigment fading and chemical and physical changes, have been revealed (Zhang et al., 2015; Palla and Barresi, 2017). Moreover, the range of biocides that can be used is limited by the Biocidal Products Regulation EU 528 (Sterflinger and Piñar, 2013). In addition, the widespread use of a limited number of antiseptics leads to the development of microbial resistance (Bastian et al., 2010; Martin-Sanchez et al., 2012). Therefore, a new generation of antiseptics that, on the one hand, exhibit targeted activity against microorganisms damaging works of art and, on the other hand, are inert to materials used in painting and non-toxic to restorers and museum visitors must be developed (Romero-Noguera et al., 2020; Alexandrova et al., 2021; Pinna, 2022; Isola et al., 2023; Zhu et al., 2023).

**Figure 1:**
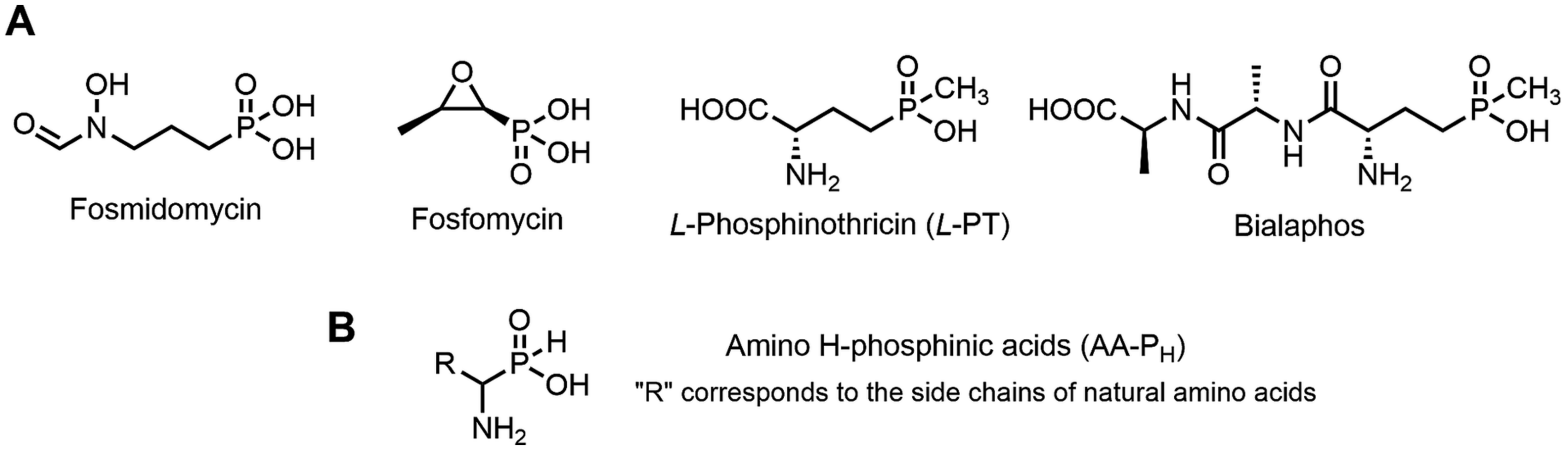
**(A)** Some biologically active natural products containing *P-C* or *C-P-C* bonds. **(B)** General structure of amino *H*-phosphinic acids.

Some microorganisms have been shown to synthesize compounds with unusual phosphorus-carbon (*P*-*C*) bonds (Ju et al., 2015; Parkinson et al., 2019; Kayrouz et al., 2020; Shiraishi and Kuzuyama, 2021; Ju and Nair, 2022). The *P*-*C* bond is biochemically stable and can mimic a phosphate monoester, whereas the tetrahedral phosphorus-containing group with a *P*-*C* bond is a mimetic of the tetrahedral intermediate/transition state arising during carboxyl group transformations (Metcalf and Van Der Donk, 2009; Horsman and Zechel, 2017). Among these secondary metabolites with *P-C* bonds, substances with diverse biological activities have been reported. For example: (i) Fosmidomycin (Figure 1A), an antibiotic and a specific nanoM inhibitor of DXP reductoisomerase, a key enzyme in the non-mevalonate pathway of isoprenoid biosynthesis (Kuzuyama et al., 1998; Jawaid et al., 2009); and (ii) Fosfomycin (Figure 1A), an irreversible inhibitor of UDP-*N*-acetylglucosamine enolpyruvyl transferase (MurA), catalyzing a key stage of the biosynthesis of cell wall peptidoglycan, thus preventing bacterial cell division (Hendlin et al., 1969; Kahan et al., 1974).

Among the secondary metabolites with two phosphorus-carbon (*C-P-C*) bonds, *L*-Phosphinothricin (*L-*PT, Figure 1A) is a notable glutamate analog with a (*C-P-CH*_3_) group replacing the γ-carboxyl group (Metcalf and Van Der Donk, 2009). *L-*PT irreversibly inhibits glutamine synthetase, which catalyzes the ATP-dependent formation of glutamine from glutamate and ammonia (Gill and Eisenberg, 2001). Glutamine synthetase plays a key role in nitrogen assimilation, and its inhibition leads to the accumulation of toxic levels of ammonia, resulting in cell death (van Heeswijk et al., 2013). However, *L*-PT, like other aminophosphonates, poorly penetrates cells and is practically important as a tripeptide of *L-*PT, Bialaphos (*L*-alanyl-*L*-alanyl-*L*-phosphinothricin), which is among the top commercial herbicides (Leason et al., 1982). This naturally occurring tripeptide yields *L*-PT, an inhibitor of glutamine synthetase, upon cleavage in the cell. Bialaphos has excellent activity against *E. coli* (Hörömpöli et al., 2021). Recently, it was shown that Bialaphos and the dipeptide *L*-Leucyl-*L*-PT are effective against clinical isolates of *Klebsiella pneumoniae*, which are resistant to more than 20 commercial antibiotics of different classes (Demiankova et al., 2023).

Aminoalkyl *H*-phosphinic acids (AA-P_H_, Figure 1B), containing carbon-phosphorus-hydrogen (*C-P-H*) bonds, have been studied significantly less compared to aminophosphonates, but unlike the latter, they penetrate microorganisms and cells and have different biological activities. It is known that AA-P_H_ can undergo substrate-like enzymatic transformations, yielding metabolites with a *C-P-H* bond, which are biologically active, and the targets of these metabolites are different from those of parent AA-P_H_ (Table 1). These and other intracellular transformations of AA-P_H_ are essential for understanding the biological effects of AA-P_H_ and for reducing the risk of developing drug resistance.

**Table 1:** Substrate-like enzymatic transformations and metabolic targets of some amino *H*-phosphinic acids.

Substrate	Enzyme	Product	Target	References
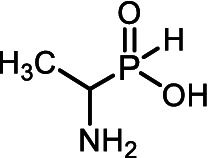 Ala-P_H_	Alanine aminotransferase	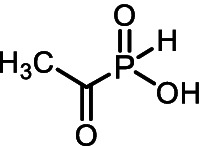 Pyr-P_H_	Inhibition of Ac-CoA biosynthesis	Laber and Amrhein (1987)
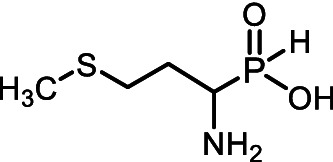 Met-P_H_	S-Adenosyl-methionine synthetase	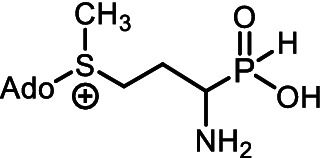 SAM-P_H_	Inhibition of some methyltransferase reactions	Rudenko et al. (2024)
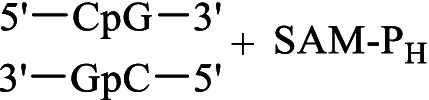	DNA methyltransferase Dnmt3a	Methylation of the CpG site 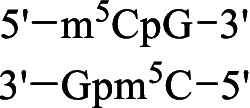		Filonov et al. (2023), Filonov et al. (2025)
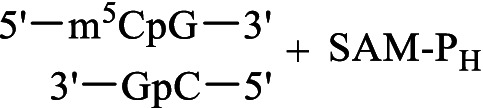	DNA methyltransferase Dnmt1	No methylation of the CpG site 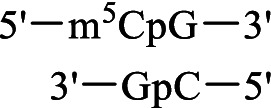		
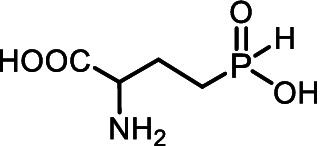 Glu-γ-P_H_	Glutamate decarboxylase	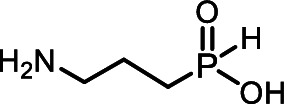 GABA-P_H_	Pleiotropic effects on metabolism	De Biase et al. (2020)
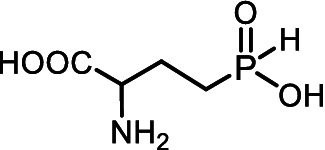 Glu-γ-P_H_	Glutamate dehydrogenase	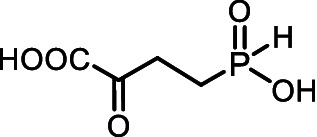 α-KG-γ-P_H_		Filonov et al. (2024)
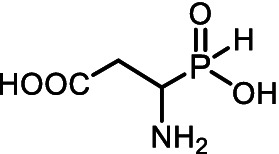 Asp-α-P_H_	Aspartate aminotransferase	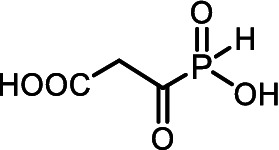 OAA-P_H_		Khurs et al. (1989)

The *H*-phosphinic analog of alanine (Ala-P_H_, Table 1) effectively inhibited anthocyanin synthesis in buckwheat hypocotyls and the growth of *K. pneumoniae* because it is transaminated intracellularly into the *H*-phosphinic analog of pyruvate (Pyr-P_H_), one of the most efficient inhibitors of pyruvate dehydrogenase (Laber and Amrhein, 1987). Ala-P_H_ bleaches the mycelium of *Pyricularia oryzae* due to the intracellular formation of Pyr-P_H,_ which decreases the levels of Ac-CoA and malonyl-CoA—precursors of fungal melanin (Zhukov et al., 2004b). The *H*-phosphinic analog of methionine (Met-P_H_, Table 1) inhibits the growth of L1210 cells, and the *H*-phosphinic analog of *S*-adenosylmethionine (SAM-P_H_, Table 1) was detected in these cells (Khomutov et al., 2000). Met-P_H_ has superior fungicidal activity in field trials (equal to the Japanese fungicide, Fujione®) against rice blast disease caused by *P. oryzae*; however, the molecular mechanisms underlying this activity have not been studied (Zhukov et al., 2004a). The distal *H*-phosphinic analog of glutamate (*L*-Glu-γ-P_H_, Table 1) is a naturally occurring compound of this class (Murakami et al., 1992; Ju et al., 2015) and has antibacterial activity comparable to that of ampicillin against *E. coli* (De Biase et al., 2020). The metabolomic and proteomic analyses of *E. coli* treated with *L*-Glu-γ-P_H_ demonstrated diverse effects of this glutamate analog (Giovannercole et al., 2024). Finally, *L*-Glu-γ-P_H_ has negligible toxic effects when administered to rats and mice (Takara et al., 1982).

**Figure 2:**
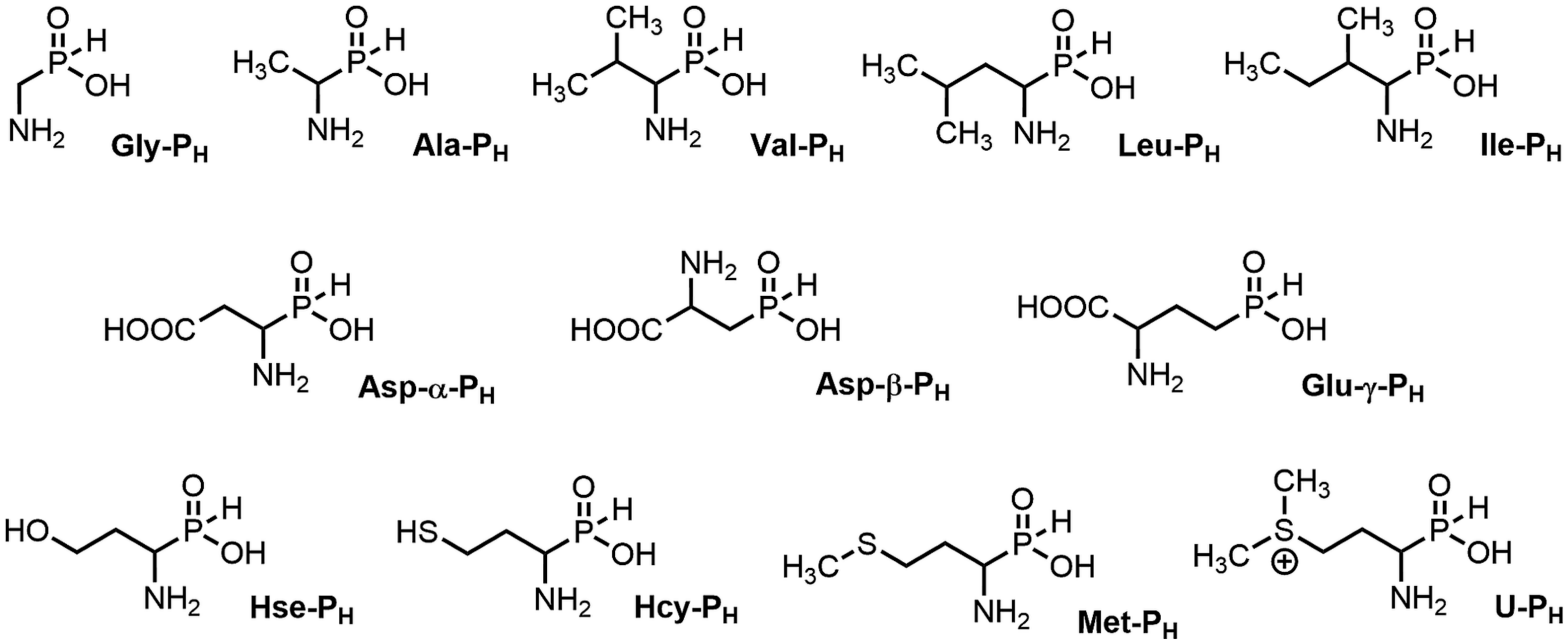
Compounds studied in the current work as antiseptics protecting paintings from biodeterioration: *H*-phosphinic analogs of glycine—Gly-P_H_, valine—Val-P_H_, leucine—Leu-P_H_, isoleucine—Ile-P_H_, aspartic acid—Asp-α-P_H_, β-aspartic acid—Asp-β-P_H_, glutamic acid—Glu-γ-P_H_, homoserine—Hse-P_H_, homocysteine—Hcy-P_H_, methionine—Met-P_H_, and vitamin U (*S*-methylmethionine)—U-P_H_.

All of the above prompted us to study the antifungal activity of 12 *H*-phosphinic analogs of natural amino acids (Figure 2) against a panel of test cultures of mold fungi that destroy painting materials. The target of these AA-P_Hs_ will be determined by the structure of the side chain of the analog. Mold fungi were isolated from the paintings exhibited in the halls of the ancient Russian paintings of the State Tretyakov Gallery, Moscow (Zhgun et al., 2020). The sensitivity of these strains to traditional antiseptics used to protect paintings, such as benzalkonium chloride (BAC) or sodium pentachlorophenolate (NaPCP), has been previously studied, and some representatives of the genera *Aspergillus* and *Cladosporium* have been found to be resistant to these compounds (Alexandrova et al., 2024; Ermolyuk et al., 2024). All studied AА-P_Hs_ demonstrated varied antifungal activity in experiments on agarized Czapek–Dox medium. Ala-P_H_, Met-P_H_, Asp-α-P_H_, and the *H*-phosphine analog of valine (Val-P_H_) exhibited the best activity. In the experiments on mock layers with sturgeon glue, Met-P_H_ was more active than traditional antiseptics (BAC and NaPCP) and overcame the drug resistance of *Aspergillus* and *Cladosporium* strains. This is the first application of water-soluble non-toxic *H*-phosphinic analogs of amino acids to prevent biodeterioration of painting materials.

## Materials and methods

### Materials

1-Aminomethyl-*H*-phosphinic acid (Gly-P_H_) was synthesized as described by Grobelny (1989). 1-Aminoethyl-*H*-phosphinic acid (Ala-P_H_), l-amino-2-methylpropyl-*H*-phosphinic acid (Val-P_H_), l-amino-3-methylbutyl-*H*-phosphinic acid (Leu-P_H_), l-amino-2-methylbutyl-*H*-phosphinic acid (Ile-P_H_), and 1-amino-3-hydroxypropyl-*H*-phosphinic acid (Hse-P_H_) were prepared following Baylis et al. (1984); 1-amino-3-methylthiopropyl-*H*-phosphinic acid (Met-P_H_), 1-amino-3-thiopropyl-*H*-phosphinic acid (Hcy-P_H_), and l-amino-3-(dimethylthionia)propyl-*H*-phosphinic acid (U-P_H_) were prepared as described in Rudenko et al. (2024); l-amino-2-carboxyethyl-*H*-phosphinic acid (Asp-α-P_H_), and 2-amino-2-carboxyethyl-*H*-phosphinic acid (Asp-β-P_H_) were synthesized according to Khomutov et al. (1996); and 3-amino-3-carboxypropyl-*H*-phosphinic acid (Glu-γ-P_H_) was prepared following Khomutov et al. (2016).

Commercial antiseptic compounds used to protect painting materials: sodium pentachlorophenolate (NaPCP) was purchased from IndiaMART, India, and benzalkonium chloride (BAC, also known as alkyldimethylbenzylammonium chloride and by the trade name Katamin AB) was purchased from Neochemax, Russia. Materials for crafting mock layers: wooden plank—LLC Mytishchi Woodworking Plant (Mytishchi, Russia); canvas—LLC Belarusian Len-Ivanovo (Ivanovo, Russia); chalk—JSC Shebekinsky Chalk Plant (Shebekino, Belgorod region, Russia); sturgeon glue—LLC Condor (Moscow, Russia).

### Strains

To determine the antifungal activity of the studied amino *H*-phosphinic acids, a panel of 11 strains of filamentous fungi, previously isolated from exhibits and in the halls of ancient Russian paintings in the State Tretyakov Gallery (STG-strains), was used (Zhgun et al., 2020). *Aspergillus versicolor* STG-25G (SRX7729174; MK260015.1) and *Ulocladium* sp. AAZ-2020a STG-36 (MW590700.1; SRX7729176) were isolated from the icon “The Church Militant” (dated 1550s). *Cladosporium halotolerans* STG-52B (SRX7729178; MK258720.1) was isolated from a bust fragment of the statue “Holy Great Martyr George the Victorious” (1,464, limestone, tempera). *Aspergillus creber* STG-57 (SRX7729151; MK266993.1) was isolated from the icon “Holy Great Martyr Demetrius of Thessaloniki” (dated 16th century). *Aspergillus versicolor* STG-86 (SRX7729182; MK262781.1), *Aspergillus creber* STG-93 W (SRX7729186; MW575292.1), *Cladosporium parahalotolerans* STG-93B (SRX7729188; MK262909.1), and *Simplicillium lamellicola* STG-96 (SRX7729192; MK262921.1) were isolated from the surfaces of hall No. 61. *Microascus paisii* STG-103 (SRX7729190; MW591474.1) was isolated from the hall No. 57. *Aspergillus protuberus* STG-106 (SRX7729192; MK268342.1) was isolated from the hall No. 56. *Penicillium chrysogenum* STG-117 (MW556011.1) was isolated from the surface of the icon “Prophet Solomon” (dated 1731).

### Cultivation of fungal strains on agarized nutrient media and growth inhibition assay

Fungal cultures were cultivated on slant agarized Czapek–Dox (CDA) medium, as described previously (Hyvönen et al., 2020). To determine the toxic effect of AA-P_H_ on mycelial growth, fungal cells were collected from agar slants; 3 μL of fungal spore suspension (5 × 10^5^ CFU/mL) was inoculated as drops onto the center of Petri dishes containing CDA medium supplemented with the addition of AА-P_H_, BAC, or NaPCP at a concentration of 0.7 mM or without any additives (control). A drop of fungal cells was absorbed into the agar, which made it possible to observe the radial growth of the mycelium from the center of the Petri dishes or to record its absence in cases of 100% inhibition. To obtain an agar medium with additives, the CDA medium was autoclaved at 120°С for 1 h and cooled to 60–65 °С. Then, AA-P_H_, BAC, and NaPCP were sterilized by filtration (pore diameter 0.22 μm) and added to agar to reach a final concentration of 2.5 mM; 22.5 mL of agar was poured into each 90 mm Petri dish. Incubation was carried out for 40 days at 26°С. The inhibitory effect was measured every 5 days and evaluated by the ratio of mycelial growth on CDA medium with the relevant addition to the mycelial growth in the control. Fungal growth inhibition (FGI) was determined using the following formula: FGI % = [(D_c_–D_t_)/D_c_] × 100, where D_c_ indicates the colony diameter in the control set, and D_t_ indicates the colony diameter in the treated set. The data were measured in triplicate and repeated at least three times.

### Crafting of mock layers

The canvas was soaked in a 10% solution of sturgeon glue and placed on 8 mm thick birch boards. The materials were dried for 24 h at room temperature, and then three layers of gesso (a 7% solution of sturgeon glue and sifted chalk, 1:3 by volume) were applied and dried for 24 h, and the surface was leveled with sandpaper to prepare the workpieces. To introduce the studied compounds (AА-P_H_ and antiseptics currently used to protect paintings) into the composition of the mock layers, so-called active mixtures were first prepared. For this purpose, 30 mM of compounds were added to a 7% solution of sturgeon glue, freshly prepared at 55–60 °C, to obtain active mixtures; to obtain the negative control, nothing was added to the sturgeon glue. The additives were AA-P_H_ (individual compounds or cocktails based on them are listed in Table 2), BAC, and NaPCP. Seven types of these active mixtures were applied in three layers on the prepared workpieces to create seven types of mock layers.

**Table 2:** Compounds and their concentrations are used to craft mock layers.

Mock layer No.	Feature	Concentration of added compounds
I	Cocktail of AА-P_H_ (components A-D)	7.5 мМ Gly-P_H_ 7.5 мМ Met-P_H_ 7.5 мМ Asp-α-P_H_ 7.5 мМ Asp-β-P_H_
II	Cocktail component A	30 мМ Gly-P_H_
III	Cocktail components B/C	15 мМ Asp-α-P_H_ 15 мМ Asp-β-P_H_
IV	Cocktail component D	30 мМ Met-P_H_
V	Positive control	30 мМ BAC
VI		30 мМ NaPCP
VII	Negative control	No additions

### Fourier-transform infrared spectroscopy of selected materials and mock layers

Infrared spectra of mock layers containing sturgeon glue with and without AA-P_H_ and standard antiseptics were acquired using a Nicolet™ iS50 Fourier transform infrared (FTIR) spectrometer (Thermo Fisher Scientific, Waltham, MA, United States), as described previously (Zhgun et al., 2022).

### Atomic force microscopy of mock layers

The atomic force microscopy technique (AFM) was used to study the surface topography of the prepared mock layers containing sturgeon glue with and without AA-P_H_ and standard antiseptics. The NTEGRA Prima microscope (NT MDT SI, Zelenograd, Russian Federation) was used in semi-contact mode with silicon cantilevers Etalon HA FM (TipsNano, Zelenograd, Russian Federation) having resonance frequencies of 77–114 kHz and force constants of 3.5–6.0 N/m; the images were processed and analyzed using the manufacturer’s software to extract surface roughness parameters and to compare topographical features between samples. The results were processed, and the statistical parameters were calculated using the Image Analysis P9 v.3.5.0.9900 program (NT-MDT SI, Zelenograd, Russian Federation). At least five AFM images (for each of the sizes: 2 × 2, 5 × 5, 10 × 10, and 20 × 20 μm) for each sample were used to calculate the root mean square surface roughness (Sq) and peak-to-valley height (St).

### Scanning electron microscopy of mold fungus

The microstructure of the mold fungus growing on the control mock layers (without additives) was investigated using scanning electron microscopy (SEM); the images were acquired using a Carl Zeiss NVision-40 microscope (Carl Zeiss, Inc., Germany). Samples with mold fungus were collected from the control mock layers (1 month after inoculation), fixed on an aluminum objective table with conductive carbon tape, placed in a vacuum chamber of a microscope, and the operating pressure was adjusted to 5.5 × 10^−6^ mbar. An Everhart-Thornley secondary electron detector with a focal length of approximately 3.3 mm was used to study the material surfaces. In order to minimize the impact of the electron beam on the sample’s structure, its surface was scanned at a sufficiently low accelerating voltage (1 kV). Owing to the relatively low electrical conductivity of the studied specimens, the magnification was limited to 250–15,000 times.

### Determination of the antiseptic properties of amino *H*-phosphinic acids in mock layers

Mock layers (with AA-P_H_, BAC, and NaPCP and without additions) were transferred to sterile Petri dishes and saturated with 0.2 mL H_2_O/1.0 cm^3^ at 26 °C for 48 h. The sterile hydrophobic pads were used to avoid direct contact of the material with water. To determine the antiseptic properties of AA-P_H_ in mock layers, the drop-dilution method was used with some modifications, as described previously (Dumina et al., 2013; Zhgun et al., 2019). Fungal cells were collected from CDA slants with 0.9% NaCl and diluted to 5 × 10^6^ CFU/mL (designated as dilution 10^−1^); sequential tenfold dilutions were performed in 0.9% NaCl. Subsequently, 3 μL of each cell suspension at concentrations of 5 × 10^6^, 5 × 10^5^, and 5 × 10^4^ CFU/mL was inoculated onto presaturated mock-up layers and incubated at 26 °C for 40 days. FGI was determined as described in the above section, Cultivation of Fungal Strains on Agarized Nutrient Media and Growth Inhibition Assay.

## Results

### Effect of *H*-phosphinic analogs of amino acids (AA-P_H_) on the growth of fungal cells on agarized Czapek–Dox medium

To determine the potential of AA-P_H_ as a new antiseptic, its effect on the growth of fungi that destroy paint and varnish materials was first studied. The target fungi were identified and isolated from the surfaces of artworks in the collections of the State Tretyakov Gallery (Moscow) (Zhgun et al., 2020). A total of 11 strains were used as test cultures; 5 belonged to the genus *Aspergillus* (*A. versicolor* STG-25G, *A. creber* STG-57, *A. versicolor* STG-86, *A. creber* STG-93 W, and *A. protuberus* STG-106); 2 belonged to the genus *Cladosporium* (*C. halotolerans* STG-52B; and *C. parahalotolerans* STG-93B), as well as one representative of each of the genera *Penicillium* (*P. chrysogenum* STG-117), *Simplicillium* (*S. lamellicola* STG-96), *Microascus* (*M. paisii* STG-103), and *Ulocladium* (*Ulocladium* sp. AAZ-2020a STG-36). This particular panel of fungi that destroys painting materials was chosen because these microorganisms are the dominant representatives of the microbiome of the Tretyakov Gallery, and the data obtained may have practical significance. Recently, the effects of two classes of biocides, such as alkyl nucleosides and chitosans (Alexandrova et al., 2022; Zhgun et al., 2022; Ermolyuk et al., 2024), were examined using this set of test cultures.

**Figure 3:**
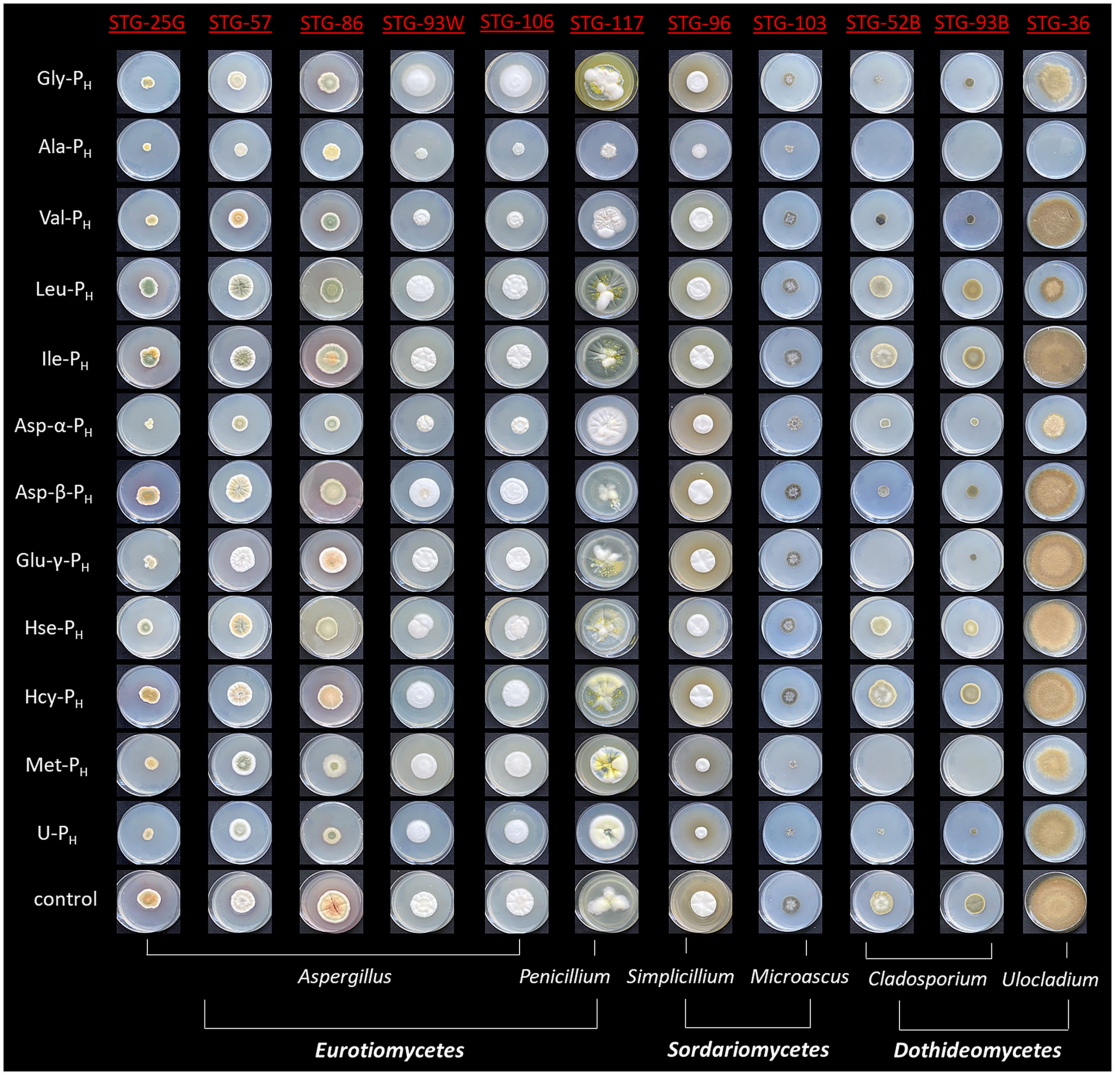
The characteristic phenotype of fungal strains of the Tretyakov Gallery on Petri dishes on Czapek–Dox agar medium (cultivation for 20 days) with the addition of *H*-phosphinic analogs of some natural amino acids (2.5 mM) or without additives (control).

All tested AA-P_H_ compounds demonstrated inhibitory effects on the mycelial growth of a panel of mold fungi on Czapek–Dox agar medium, with the degree of inhibition varying depending on the structure of the side chain of the analog and the fungal species (Figure 3).

**Figure 4:**
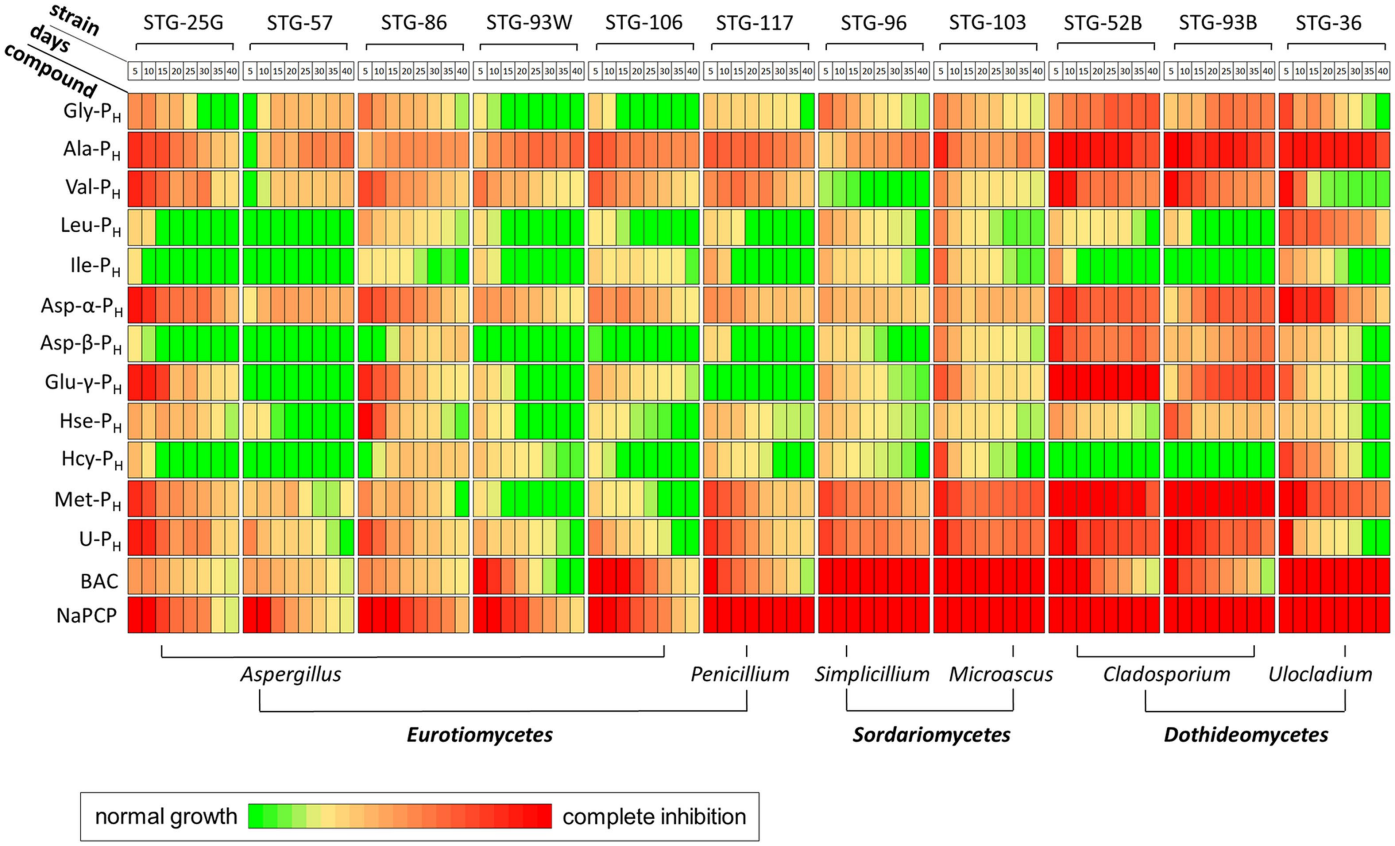
Effects of *H*-phosphinic analogs of natural amino acids and standard antiseptics on the growth of fungal strains of the Tretyakov Gallery on Czapek–Dox agar medium. Green, normal growth (no inhibition); red, complete inhibition. The degree of fungal growth inhibition was normalized to that of the control. BAC, benzalkonium chloride; NaPCP, sodium pentachlorophenolate. Data were acquired at 5, 10, 15, 20, 25, 30, 35, and 40 days after inoculation.

To quantify the effects of AA-P_H,_ the dynamics of fungal growth inhibition (FGI) were studied for 40 days after inoculation of the test fungi on the experimental and control media. Fungal growth was analyzed every 5 days (Figure 4).

The effectiveness of the studied compounds significantly depends on the structure of the side radical of AA-P_H_. Among the tested compounds, Ala-P_H_ and Asp-α-P_H_ exhibited the best antifungal activity, inhibiting the growth of all the studied strains (Figure 4). Moreover, Ala-P_H_ completely inhibited the growth of STG-36, STG-52B, and STG-93B throughout the cultivation period (Figure 4). Among the studied compounds, Val-P_H_, Met-P_H_, and U-P_H_ showed high activity, inhibiting the growth of more than 80% of the test cultures throughout the experimental period. Simultaneously, Gly-P_H_, Asp-β-P_H_, and Glu-γ-P_H_ exhibited high activity, mostly against *Cladosporium* (Figure 4).

Among the analogs of aliphatic amino acids, Ala-P_H_ exhibited the highest activity, followed by Val-P_H_. Interestingly, among the *H*-phosphinic analogs of branched-chain amino acids, only Val-P_H_, but not Leu-P_H_ and Ile-P_H,_ exhibited good activity (Figure 4). This structure–activity relationship confirms that the nature of the side radical of the *H*-phosphinic amino acid analogs is important for antifungal activity. The highest activity of Ala-P_H_ is most likely related to its intracellular transamination, yielding the *H*-phosphinic analog of pyruvate—one of the most effective inhibitors of pyruvate dehydrogenase (PDH). This has been for buckwheat hypocotyls (Laber and Amrhein, 1987) and *Pyricularia oryzae* (Zhukov et al., 2004b). In the first case, inhibition of PDH resulted in the inhibition of Ac-CoA-dependent anthocyanin biosynthesis, and in the second, melanin biosynthesis was inhibited, and the fungi’s mycelium became colorless.

A comparison of the activities of two analogs of aspartic acid, which differ in the position of the *H*-phosphinic substituent (α and β positions), is of interest. Asp-α-P_H_ is more active than Asp-β-P_H_ against all fungi studied, and only against STG-52B are their activities comparable (Figure 4). The *H*-phosphinic analog of glutamate (Glu-γ-P_H_) has a similar inhibition dynamic profile to Asp-α-P_H_ but has slightly weaker activity (Figure 4).

Methionine is one of the key compounds in sulfur metabolism. This may explain why its *H*-phosphinic analog (Met-P_H_) was among the most active AA-P_H_ (Figure 4). Homocysteine is a direct metabolic precursor of methionine, and we assumed that Hcy-P_H_ may have fungicidal activity close to that of Met-P_H_. However, this assumption was not confirmed, as the effect of Hcy-P_H_ on the growth of fungal cells was significantly weaker than that of Met-P_H_ (Figure 4). It is possible that in fungal cells, Hcy-P_H_ is not metabolized to Met-P_H_ and is, therefore, less effective in the metabolic fluxes of the cell. In this regard, the data obtained for the analog of another important compound involved in sulfur metabolism, vitamin U, is of interest. The activity profile of U-P_H_ against a panel of test cultures was similar to that of Met-P_H_. The dynamics of inhibition of various fungal strains were comparable for both analogs; however, Met-P_H_ exhibited somewhat higher activity.

Overall, AA-P_H_ most effectively inhibited the growth of fungi belonging to the classes *Dothideomycetes* and *Sordariomycetes*, whereas some representatives of the class *Eurotiomycetes* belonging to the genus *Penicillium* showed moderate resistance to AA-P_H_ (Figure 4). The most resistant to AA-P_H_ were fungi of the genus *Aspergillus*. Among *Aspergillus* strains, the most resistant to AA-P_H_ were strains *A. creber* STG-57, *A. creber* STG-93 W, and *A. protuberus* STG-106. However, when considering the endpoint of cultivation on day 40, the STG-36 strain was able to completely overcome the toxic effect of all AA-P_H,_ except for Ala-P_H_, Asp-α-P_H_, and Met-P_H_. At this cultivation period, the STG-93 W and STG-106 strains also completely overcame the toxic effects of 9 out of 12 AA-P_H_ studied. STG-93 W remained sensitive to Ala-P_H_, Val-P_H_, and Met-P_H_, and STG-106 remained sensitive to Ala-P_H_, Val-P_H_, and Asp-α-P_H_.

### Effect of AA-P_H_ on the pigmentation of *Penicillium chrysogenum* STG-117 on agarized Czapek–Dox medium

**Figure 5:**
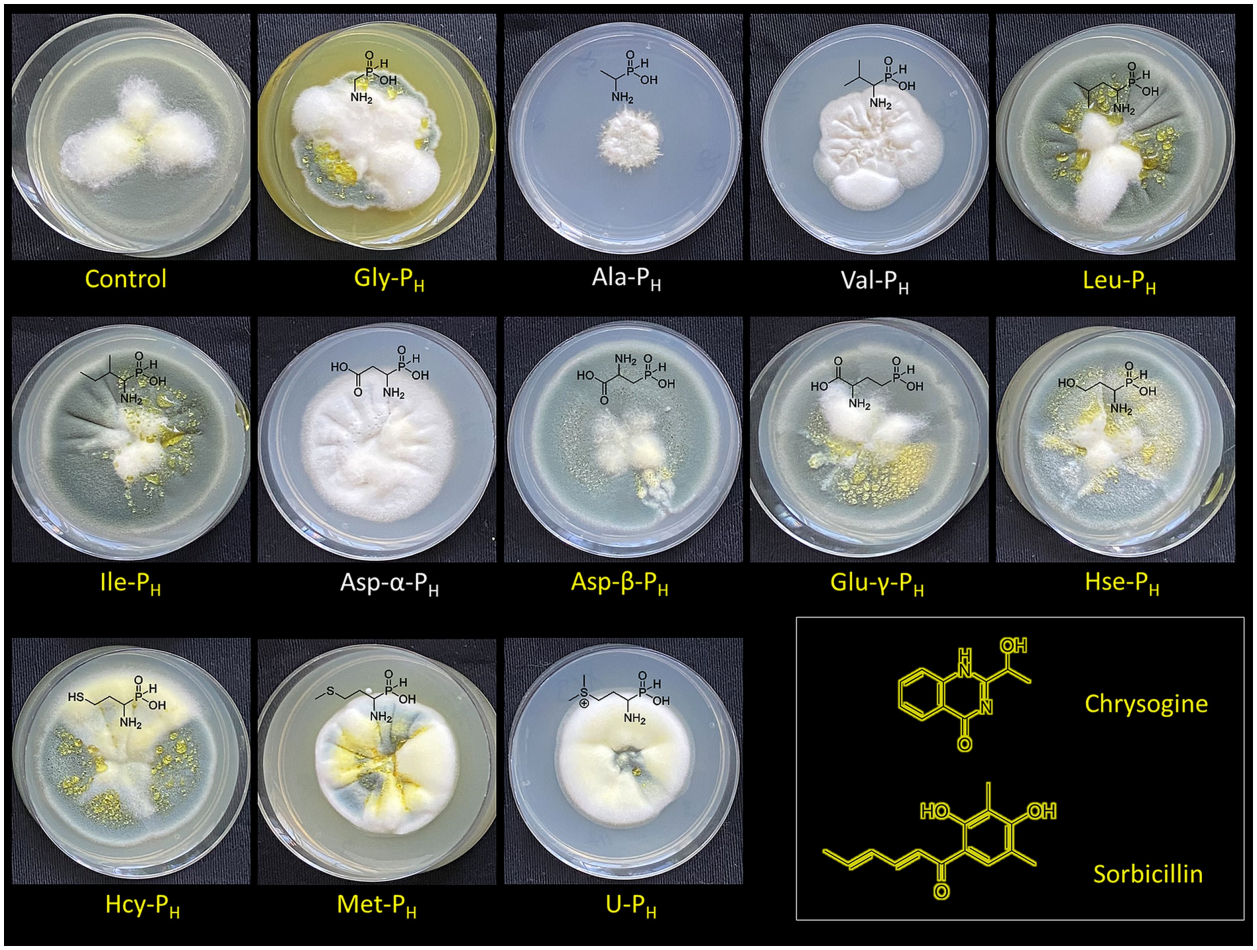
Pigmentary phenotype of *Penicillium chrysogenum* STG-117 after cultivation for 20 days on Czapek–Dox agar medium with the addition of different *H*-phosphinic analogs of natural amino acids (2.5 mM) or without additives (control). Structural formulas of chrysogine and sorbicillin, which determine the color of *P. chrysogenum,* are shown. The addition of Ala-P_H_, Asp-α-P_H_, and Val-P_H_ resulted in bleaching of *P. chrysogenum* colonies.

It has been shown that during the cultivation of *Penicillium* strains on agarized nutrient media, in the period after the transition from the trophophase to the idiophase stage, a characteristic greenish-yellow color develops. This pigmentation is due to the biosynthesis of chrysogenin and sorbicillin (secondary metabolites), which color the fungal colonies, exudates, and agarized medium (Demain, 1986; Martín et al., 2012). Supplementation with Ala-P_H_, Asp-α-P_H,_ and Val-P_H_ resulted in the loss of characteristic pigmentation in *P. chrysogenum* STG-117, which may be associated with inhibition of acetyl-CoA and malonyl-CoA biosynthesis, precursors for fungal melanin and other polyketide pigments (Figure 5).

The effects of some AA-P_H_ on the morphology and pigmentation of *P. chrysogenum* STG-117 were somewhat unexpected and indicate that these compounds differentially affect the secondary metabolic system of this fungus. The bleaching activity of AA-P_H_ depends on the radical structure, and only Ala-P_H_, Val-P_H_, and Asp-α-P_H_ have such potential (Figure 5). The possible effects of these AA-P_H_ on the biosynthesis of chrysogenin and sorbicillin in *P. chrysogenum* STG-117 are related to the inhibition of Ac-CoA/HS-CoA biosynthesis and are discussed in the Discussion section.

### Crafting of mock layers containing AA-P_H_ or control antiseptics

In order to develop an effective antiseptic that is not harmful to painting materials, we prepared a cocktail from several AA-P_H_ (Table 2). These AA-P_H_ showed high activity in standard microbiological media, CDA (Figure 4), and are the analogs of amino acids involved in key metabolic hubs. We assumed that this approach may lead to an antiseptic with higher antifungal activity because of the simultaneous effect on independent metabolic targets. To understand the contribution of individual components of the cocktail, we also prepared a series of mock-ups based on them. A practically essential antiseptic must have minimal impact on the physical and chemical properties of the material. When antiseptics are added to painting materials, their concentration does not typically exceed 1%. For example, BAC (1%) or NaPCP (1%) was used in studies with mock-ups; i.e., the antiseptic concentrations were 29 mM and 34 mM, respectively (Zhgun et al., 2020, 2022; Avdanina et al., 2024, 2025). To obtain an accurate comparison of the effects of AA-P_H_ and standard antiseptics, all the studied compounds were used at a concentration of 30 mM. When studying the effects of the compound cocktail, an equal amount of components were used to achieve a total concentration of 30 mM.

We included Gly-P_H_ in the cocktail because it showed significant activity in CDA medium and because glycine is essential for multiple metabolic pathways, including glutathione synthesis and one-carbon metabolism (Meléndez-Hevia and De Paz-Lugo, 2008; Alves et al., 2019; Hong et al., 2020). Exogenous administration of glycine can stimulate fungal growth and the production of antimicrobial compounds (Wu et al., 2015).

Another important metabolic hub is associated with aspartic acid because of its centrality in essential metabolic transformations, such as the TCA cycle, nucleotide metabolism, hormone biosynthesis, etc. (Azevedo et al., 2006; Sullivan et al., 2015; Lei et al., 2022; Dumina and Zhgun, 2023; Holeček, 2023). We included both Asp-α-P_H_ and Asp-β-P_H_ in the cocktail for alternative inhibition of target enzymes.

The third hub targeted by the developed AA-P_H_ cocktail is related to sulfur-containing amino acid metabolism. Methionine occupies a central role in metabolism and growth control in fungi (Garbe and Vylkova, 2019; Shrivastava et al., 2021; Rząd et al., 2024). Methionine and its derivatives induce anabolic programs and control various processes integral to metabolism, such as one-carbon metabolism, nucleotide synthesis, and redox balance (Walvekar and Laxman, 2019; Lauinger and Kaiser, 2021). Methionine is the metabolic precursor of *S*-adenosylmethionine, which ranks second only to ATP in terms of the diversity of biochemical transformations it participates in, donating methyl groups to a wide range of biological substrates and being an important epigenetic regulator (Lee et al., 2023). In particular, *S-*adenosylmethionine is involved in the control of development, secondary metabolism, and virulence of fungi (Gerke et al., 2012; Scott et al., 2020; Scott and Amich, 2023). To regulate the enzymes of the methionine/S-adenosylmethionine hub, we chose Met-P_H_. which is enzymatically converted into the *H*-phosphinic analog of *S*-adenosylmethionine (Rudenko et al., 2024)—an original epigenetic regulator. Finally, Met-P_H_ exhibited excellent fungicidal activity under field trials against *Pyricularia oryzae* (Zhukov et al., 2004a) and had one of the best activities among all studied AA-P_H_ on the CDA nutrient medium (Figure 4).

**Figure 6:**
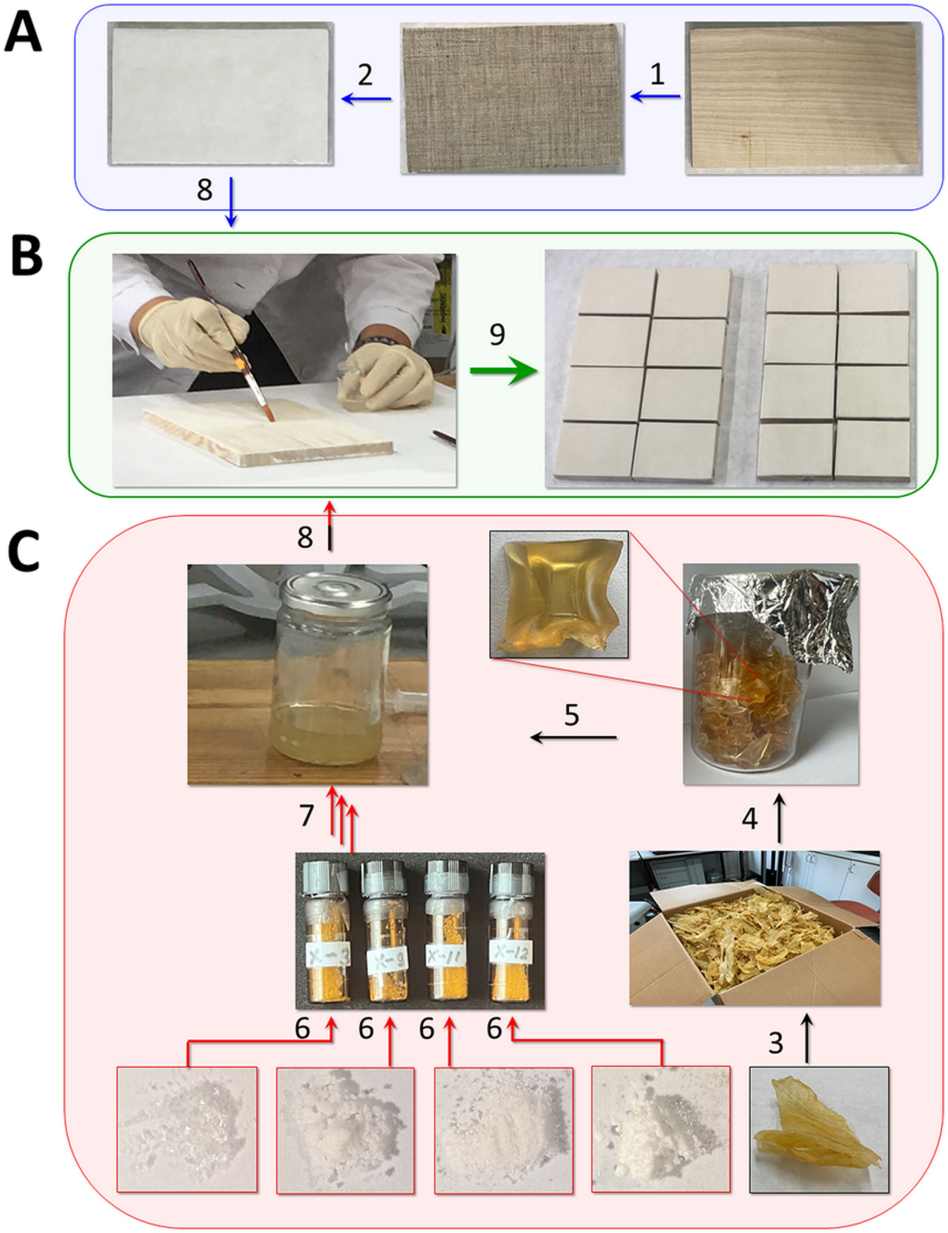
Stages of crafting mock layers. **(A)** Preparing templates for applying sturgeon glue with antiseptics; **(B)** application of sturgeon glue with antiseptics and fragmentation of mock-ups; **(C)** preparation of sturgeon glue with antiseptics. Manufacturing steps: 1: overlay of canvas (pavoloka) on wood (birch); 2: overlay of levkas (ground) and surface sanding; 3: production of layered material from sturgeon; 4: obtaining tableted dry raw materials for the preparation of sturgeon glue solutions; 5: preparation of an aqueous solution of sturgeon glue of the required concentration; 6: chemical synthesis of *H*-phosphinic analogs of amino acids; 7: addition of *H*-phosphinic analogs of amino acids to an aqueous solution of sturgeon glue; 8: application of sturgeon glue with antiseptics on templates; and 9—fragmentation of obtained mock layers.

A series of mock layers was prepared in accordance with a previously developed procedure (Zhgun et al., 2020). All tested compounds were added at a total concentration of 30 mM, as it has previously been shown that the addition of standard antiseptics (sodium pentachlorophenolate, NaPCP, or benzalkonium chloride, BAC) at this concentration does not yet have a significant effect on the properties of the materials themselves but effectively protects against biodeterioration (Zhgun et al., 2022). Therefore, the AA-P_H_ cocktail consisted of 7.5 mM Gly-P_H_, 7.5 mM Asp-α-P_H_, 7.5 mM Asp-β-P_H_, and 7.5 mM Met-P_H_ (mock layer I). The mock layers with individual components of the cocktail contained mock layer II—30 mM Gly-P_H_ (component A), mock layer III—15 mM Asp-α-P_H_ and 15 mM Asp-β-P_H_ (components B/C), and mock layer IV—30 mM Met-P_H_ (component D). The mock layers with standard antiseptics contained 30 mM BAC (mock layer V) and 30 mM NaPCP (mock layer VI). In addition, we prepared a set of mock-ups without additives for control tests (mock layer VII). The main stages of fabricating the mock layers are shown in Figure 6.

Mock layers were first characterized in terms of the impact of the introduced additives on the spectral and surface properties of the materials and were then used in experiments on infection with fungal test cultures.

### Fourier-transform infrared spectroscopy analysis of the mock layers with AA-P_H_

**Figure 7:**
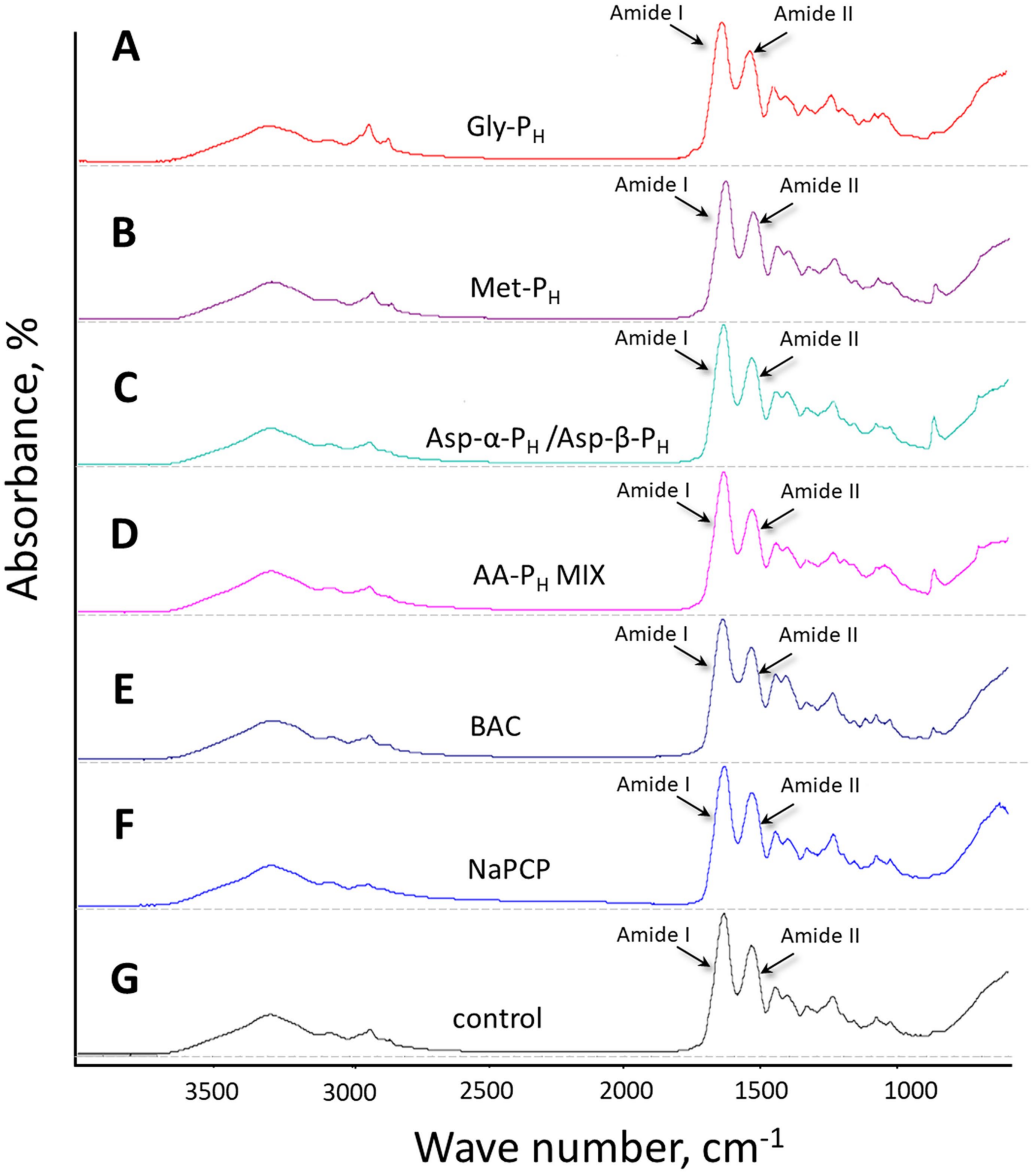
Fourier-transform infrared spectra of mock layers with the additions of AA-P_H_ and standard antiseptics. **(A)** Gly-P_H_; **(B)** Met-P_H_; **(C)** Asp-α-P_H_ + Asp-β-P_H_; **(D)** AA-P_H_ MIX, a cocktail of Gly-P_H_ + Met-P_H_ + Asp-α-P_H_ + Asp-β-P_H_; **(E)** benzalkonium chloride (BAC); **(F)** sodium pentachlorophenolate (NaPCP). **(G)** FTIR spectra of the mock layer without additives (control).

Fourier-Transform Infrared (FTIR) spectroscopy was used to assess the changes in the chemical structure of painting materials caused by the studied compounds. FTIR spectra of mock layers with AA-P_H_ additives were comparable to those of control layers, indicating that the chemical composition of the glue matrix was not substantially altered by the addition of the *H*-phosphinic analogs at the concentrations used (Figure 7). The obtained data indicate that the addition of sturgeon glue: (i) 30 mM Gly-P_H_, (ii) 15 mM Asp-α-P_H_ and 15 mM Asp-β-P_H_, (iii) 30 mM Met-P_H_, or (iv) a cocktail of AA-P_H_ (7.5 mM Gly-P_H_, 7.5 mM Met-P_H_, 7.5 mM Asp-α-P_H,_ and 7.5 mM Asp-β-P_H_) did not cause changes in the IR spectra compared to the control (Figure 7). Moreover, the characteristic bands corresponding to the *P-H* bond (2300–2,400 cm^−1^), *O=P* bond (1,150–1,200 cm^−1^), and *P-O*^−^-bond (1,010–1,080 cm^−1^) were not visible (spectra A–F, Figure 7), which is apparently because of an insufficient concentration of amino *H*-phosphinic acids in the studied samples. The addition of standard antiseptics (BAC and NaPCP) to sturgeon glue also did not cause changes in the spectral characteristics of the glue that are consistent with the previously obtained data (Zhgun et al., 2020, 2022).

### Atomic force microscopy analysis of the developed mock layers with AA-P_H_

The impact of *H*-phosphinic analogs of natural amino acids added to sturgeon glue of crafted mock layers on their surface properties was studied using atomic force microscopy (AFM). The surface parameters of the samples were calculated by analyzing the AFM images (Table 3). Root mean square (RMS) roughness was used to estimate surface heterogeneities and quantify surface conditions (Gadelmawla et al., 2002). In addition to the scale of the scanned image, the height distribution can affect the value of Sq; therefore, we also added the height parameter St (the vertical distance between the highest peak and the lowest valley). Both parameters were calculated in three-dimensional (3D) form for an area of the surface instead of a single profile on the frames with a size of 10 × 10 μm.

**Table 3:** Parameters of surface roughness of the analyzed mock layers^a^.

Mock layer number	Addition	Parameter (mean value)	
		Sq^b^, nm	St^c^, nm
I	7.5 mM Gly-P_H_ + 7.5 mM Met-P_H_ + 7.5 mM Asp-α-P_H_ + 7.5 mM Asp-β-P_H_	8.3 ± 2.5	122.1 ± 27.7
II	30 mM Gly-P_H_	88.3 ± 9.5	749.8 ± 61.2
III	15 mM Asp-α-P_H_ + 15 mM Asp-β-P_H_	5.0 ± 0.6	91.1 ± 21.2
IV	30 mM Met-P_H_	19.1 ± 1.2	229.4 ± 29.8
V	30 mM BAC	10.8 ± 3.6	178.9 ± 89.1
VI	30 mM NaPCP	6.8 ± 3.5	134.6 ± 90.9
VII	–	9.4 ± 5.4	106.5 ± 49.8

a Size of scan area was 10 × 10 μm.

b Sq, root mean square roughness of the surface.

c St, the vertical distance between the highest peak and the lowest valley.

**Figure 8:**
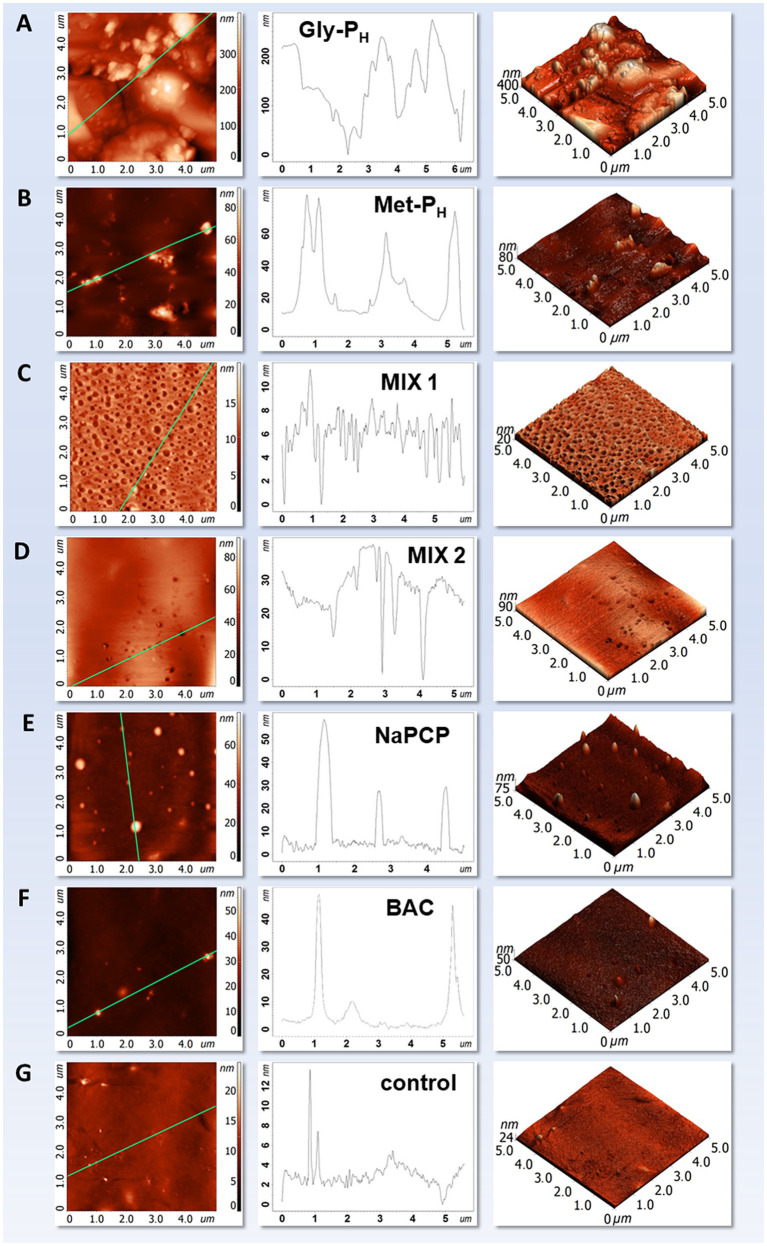
Atomic force microscopy images of mock layers. Sturgeon glue additives: **(A)** Gly-P_H_; **(B)** Met-P_H_; **(C)** MIX1, Asp-α-P_H_ + Asp-β-P_H_; **(D)** MIX2, cocktail of Gly-P_H_ + Met-P_H_ + Asp-α-P_H_ + Asp-β-P_H_; **(E)** benzalkonium chloride (BAC); **(F)** sodium pentachlorophenolate (NaPCP). **(G)** AFM image of a mock layer without additives (control). Scanning area 5 × 5 μm.

All four investigated samples with AA-P_H_ (mock layers I–IV, Table 3) formed a coating on the template surface (Figures 8A–D). This was evidenced by the difference in surface morphology compared to that of the control. For the control sample (Figure 8G), a relatively smooth surface with single inhomogeneities was observed.

Samples from mock layers with Gly-P_H_ (mock layer II) and Met-P_H_ (mock layer IV) formed nonuniform coatings consisting of large globules, which were considerably larger in the case of Gly-P_H_ (Figures 8A,B). Moreover, the addition of Gly-P_H_ forms aggregates in mock layer II with a height difference of more than 700 nm (Table 3), filling the entire surface of the template, which increased the surface roughness 9-fold compared to the control (88.3 nm and 9.4 nm for the mock layer II and control mock layer VII, respectively). On the mock layer IV surface, there were also globular particles that clumped into aggregates; however, their amount was significantly lower than that for mock layer II, and the values of the roughness parameters were twice as high as those for the control (Table 3).

The Sq and St parameters for mock layers with components C/D (mock layer III) and a cocktail of AA-P_H_ (mock layer I) were not significantly altered from the control mock layer VII, but the morphology of these materials had some peculiarities, as shown in Figures 8C,D. First, both samples filled the template to a greater extent than mock layers IV (with Met-P_H_), V (with BAC), and VI (with NaPCP) and formed films. Second, these films were more homogenous: large aggregates were absent in the AFM images, which reduced the calculated RMS roughness value, as can be seen in Table 3 for mock layer III. Third, the films had a porous structure, with the pore height of the sample from mock layer III being smaller than that of the mock layer IV but the number of pores themselves being significantly higher. The mock layers with NaPCP (Figure 8E) and BAC (Figure 8F) samples had similar surface topography, represented by single globular “drops” with varying sizes, but on average less than 60 nm, which were randomly arranged on the template surface and did not completely fill the template. One of the peculiarities of AFM analysis is that the scale of images should be considered when studying the topography of materials. Therefore, we investigated all samples at different sizes of the scanned area. The results presented in Supplementary Figure S1 show the main surface characteristics of each sample as the image scale changes.

In general, it can be concluded that among the studied mock layers with the addition of AA-P_H_, the surface properties were partially changed in mock layers I–III and were practically the same as in those of the control in mock layer IV (addition of Met-P_H_). Moreover, the effect of adding Met-P_H_ was comparable to the insignificant effect that occurred when adding BAC to sturgeon glue, which is routinely used in restoration practice to protect against biodeterioration (Table 3). The most significant changes were found for the imitation layer containing Gly-P_H_; however, we continued working with this analog to determine its antiseptic properties in the composition of sturgeon glue.

### Scanning electron microscopy analysis of micromorphology of test cultures on mock layers

**Figure 9:**
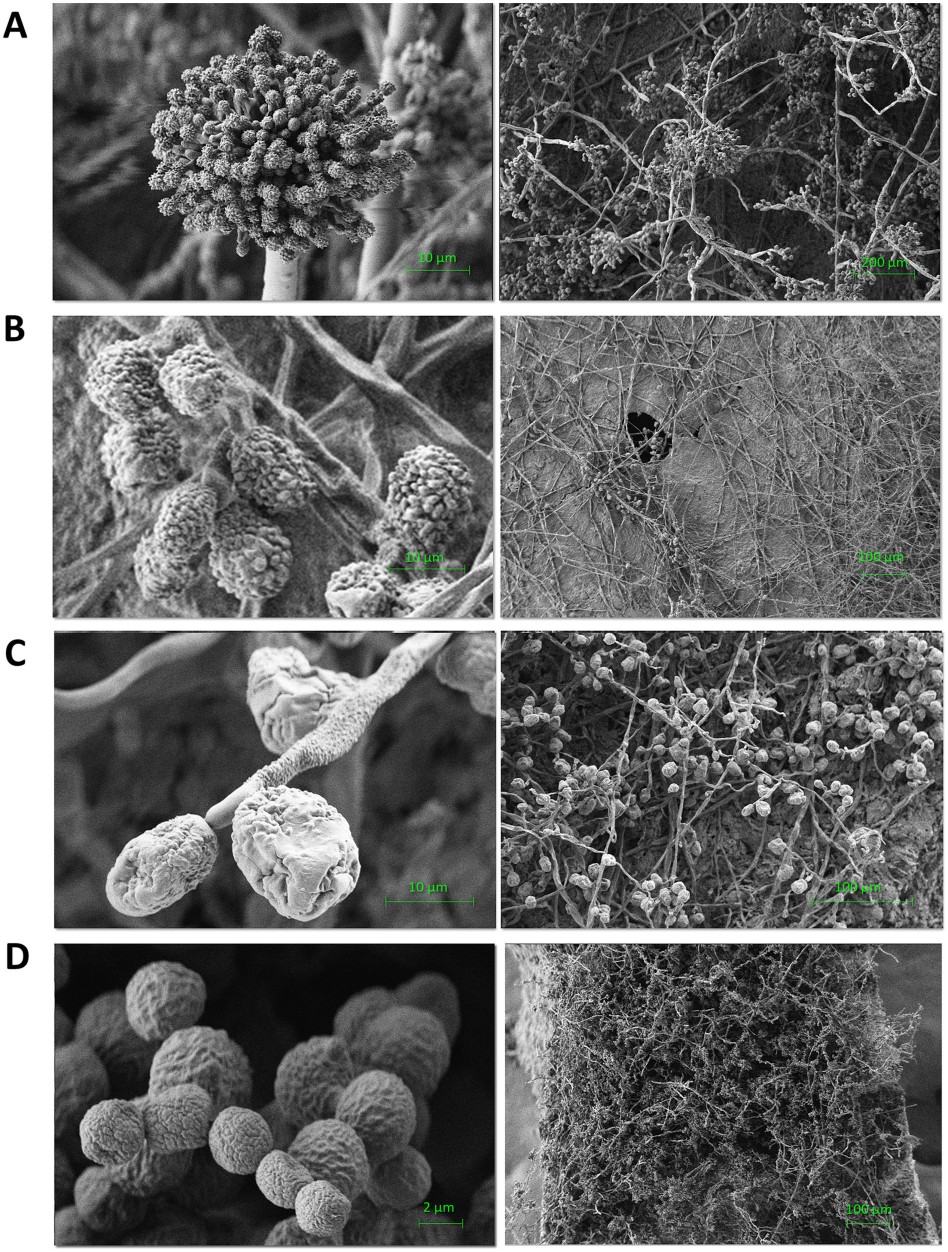
Scanning electron microscopy of STG strains grown on the control mock layer No. I (15 days after inoculation). **(A)** *Aspergillus vermicular* STG-25G; **(B)** *Simplicillium lamellicola* STG-96; **(C)** *Ulocladium* sp. AAZ-2020a STG-36; **(D)** *Cladosporium halotolerans* STG-52B.

Before studying the antiseptic activity of AA-P_H_ on the prepared mock layers, we determined how effectively the test fungal cultures grew on these mock layers. For this purpose, the micromorphology of the fungi was studied using electron scanning microscopy after inoculation of the control mock-ups (Figure 9). The resulting images demonstrate the development of a characteristic micromorphology for various types of fungi that destroy painting materials on the prepared mock layers (Figure 9). Thus, the obtained mock layer has the necessary spectral and surface characteristics, is bioavailable for the studied test cultures, and can be used to study the antiseptic properties of compounds in the AA-P_H_ family.

### Analysis of the antiseptic properties of AA-P_H_-containing mock layers

**Figure 10:**
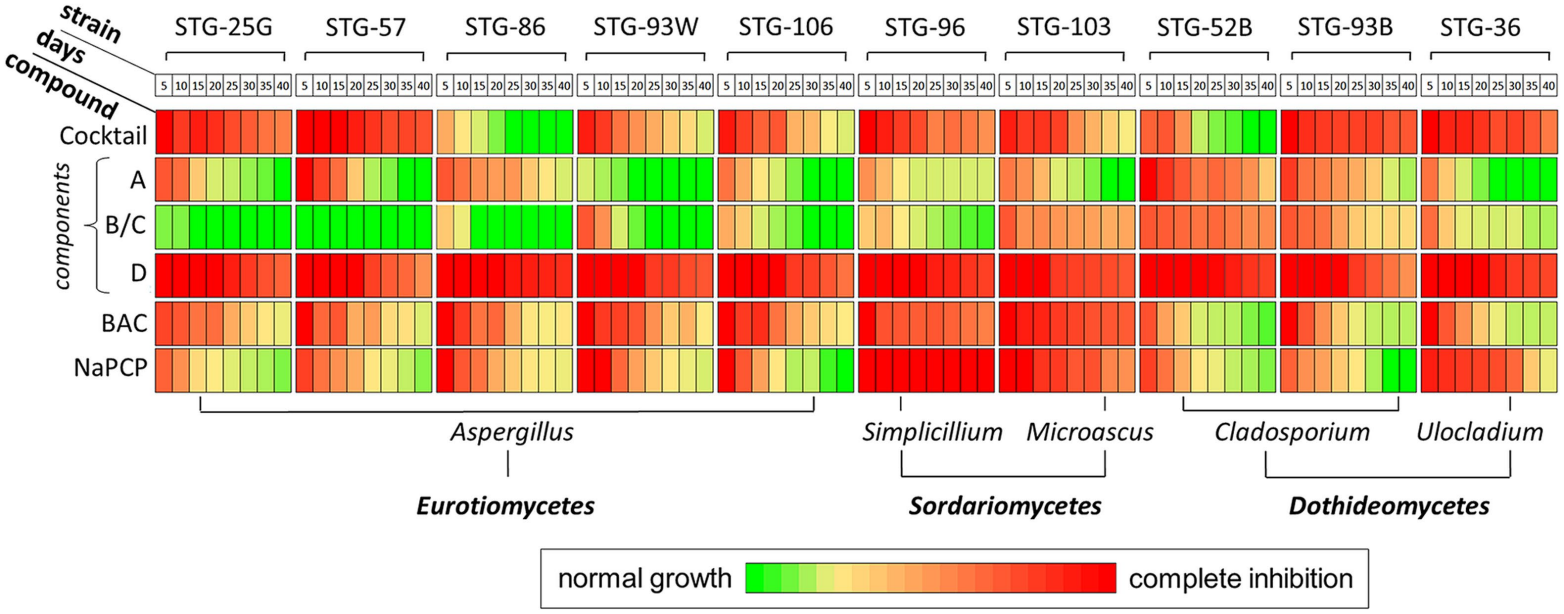
Effects of *H*-phosphinic analogs of natural amino acids and standard antiseptics on the growth of fungal strains of the Tretyakov Gallery on mock-up layers. Green, normal growth (no inhibition); red, complete inhibition. The degree of fungal growth inhibition was normalized to the control. Cocktail of AА-P_H_ (Gly-P_H_, Asp-α-P_H_, Asp-β-P_H_, and Met-P_H_); components from the cocktail of AА-P_H_: (A) Gly-P_H_, (B/C) Asp-α-P_H_ and Asp-β-P_H_, (D) Met-P_H_; BAC, benzalkonium chloride; NaPCP, sodium pentachlorophenolate. Data were acquired at 5, 10, 15, 20, 25, 30, 35, and 40 days after inoculation.

The antifungal activity of the AА-P_H_ cocktail composed of Gly-P_H_, Asp-α-P_H,_ Asp-β-P_H_, and Met-P_H_, as well as individual components of the cocktail as constituents of mock layers, was studied in comparison with the positive control (standard antiseptics BAC and NaPCP) and negative controls (mock layers without the addition of antiseptics). All antiseptics (individual compounds or the compounds in the cocktail) were added to the mock layers to obtain a final concentration of 30 mM. FGI data were obtained every 5 days after inoculation of test cultures on mock-ups for 40 days (Figure 10; Supplementary Figure S2).

The cocktail AА-P_H_ (7.5 mM Gly-P_H_, 7.5 mM Met-P_H,_ 7.5 mM Asp-α-P_H_, and 7.5 mM Asp-β-P_H_) showed significant antifungal activity against most fungal test cultures, with the exception of strains STG-86 (after 10 days of cultivation) and STG-52B (after 20 days of cultivation). This cocktail completely inhibited the growth of strains STG-25G, STG-57, STG-93B, and STG-36 over the entire duration of the experiment, and the effect of the cocktail on these strains was stronger than that of the standard antiseptics, BAC and NaPCP (Figure 10). Component A (Gly-P_H_), added to sturgeon glue at a concentration equal to the total cocktail content (30 mM), showed a significant decrease in activity against most fungal test cultures (Figure 10). However, Gly-P_H_ is not a key component of the cocktail against these fungi, and its activity was slightly weaker than that of standard antiseptics (Figure 10). Components B/C (15 mM Asp-α-P_H_ and 15 mM Asp-β-P_H_ in sturgeon glue) showed lower antifungal activity than the AА-P_H_ cocktail against the majority of the fungal strains (STG-36, STG-86, STG-93B, STG-93 W, STG-96, STG-103, and STG-106). Moreover, its activity against test cultures STG-25G and STG-57 completely disappeared. The mixture of Asp-α-P_H_ and Asp-β-P_H_ worked slightly better against STG-103 and *Cladosporium* compared to Gly-P_H_ but was significantly weaker against *Aspergillus*. Apparently, Asp-α-P_H_/Asp-β-P_H_ was also not the main component of the cocktail. However, against STG-52B, components B/C, as well as component A, showed better activity than the cocktail. It is possible that a two-fold dilution of components B/C in the cocktail leads to a decrease in activity.

Effective inhibition of the growth of all test cultures was observed when sturgeon glue contained component D (Met-P_H_) at a 30 mM concentration. The activity of Met-P_H_ was higher than that of the cocktails and standard antiseptics (Figure 10). The main active compound in the cocktail was component D, and diluting its content by four times with other AA-P_H_ led to a decrease in antifungal properties. Component D completely inhibited the growth of STG-36, STG-52B, STG-86, and STG-96 over the entire duration of the experiment. This effect was unexpected, as it was stronger than that of standard antiseptics (BAC and NaPCP), which on agar medium worked better than Met-P_H_, especially against *Aspergillus* (Figure 4). It is also not entirely clear why the effect of the cocktail on the STG-52B strain was weaker than the effect of each of its components when taken in two- or four-fold higher concentrations. It is possible that this fungus is resistant to small concentrations of the compounds of the AA-P_H_ family, with no additivity.

**Figure 11:**
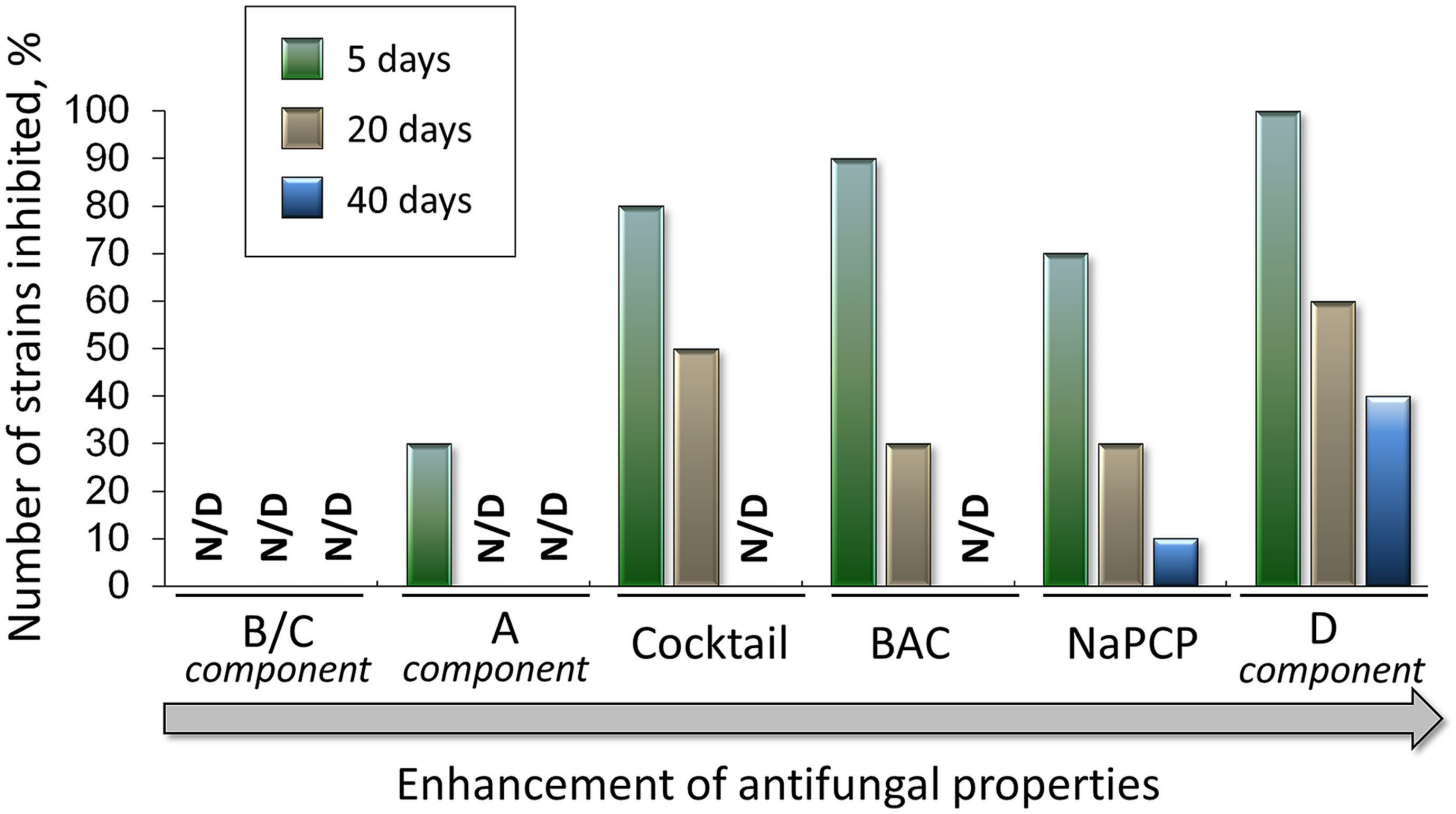
Percentage of the inhibited fungal microbiome of the Tretyakov Gallery (among the 11 studied strains) at 5, 20, and 40 days after inoculation on mock layers with the addition of selected antifungal compounds. Cocktail of AA-P_H_ (Gly-P_H_, Met-P_H,_ Asp-α-P_H_, and Asp-β-P_H_); components of the cocktail of AA-P_H_: (A) Gly-P_H_, (B/C) Asp-α-P_H_ and Asp-β-P_H_, (D) Met-P_H_; standard antiseptics: BAC, benzalkonium chloride; NaPCP, sodium pentachlorophenolate; N/D, not detected.

We quantitatively compared the effect of added AA-P_H_ on the growth inhibition of the test cultures during cultivation (Figure 11). For this, the number of completely inhibited strains at the beginning of cultivation (5th day), middle (20th day), and end of cultivation (40th day) was estimated.

The lowest activity was demonstrated by the B/C component (Asp-α-P_H_ and Asp-β-P_H_), which did not completely suppress the growth of any of the test cultures. Component A (Gly-P_H_) completely inhibited 30% of the strains at the beginning of cultivation, and the growth of these strains was subsequently observed (Figure 11). The cocktail (Gly-P_H_, Met-P_H,_ Asp-α-P_H_, and Asp-β-P_H_) completely inhibited the growth of 80% of the strains after 5 days and 50% of the strains after 20 days (Figure 11). BAC showed approximately the same activity at the beginning of cultivation—it suppressed the growth of 90% of the test cultures, but by the 20^th^ day, this value dropped to 30%, and at the end of cultivation, all strains overcame the toxic effect (Figure 11). Another standard antiseptic, NaPCP, was as active as BAC, completely suppressing the growth of 70% of fungal strains by day 5 and only 30% of strains by day 20 (Figure 11). However, NaPCP completely inhibited the growth of STG-96 toward the end of cultivation (Figure 10). Among the tested compounds and their combinations, component D (Met-P_H_) exhibited the highest activity. This *H*-phosphinic analog of methionine completely suppressed the growth of all strains at the beginning of cultivation, by 60% in the middle, and by 40% at the end of cultivation (Figure 11).

In conclusion, it can be concluded that experiments on mock layers prepared with sturgeon glue demonstrated that Met-P_H_ provided superior protection against fungal colonization compared to other AA-P_H_ compounds and standard antiseptics such as benzalkonium chloride (BAC) and sodium pentachlorophenolate (NaPCP).

## Discussion

**SCHEME 1:**
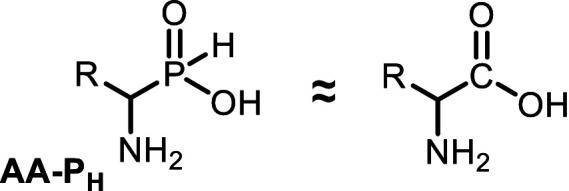
General formula of aminoalkyl *H*-phosphinic acids (AA-P_Hs_) and natural amino acids.

Many mold fungi that inhabit works of art are resistant to antiseptics and external environmental conditions (de Paiva Carvalho et al., 2018; El Jaddaoui et al., 2023; Gadd et al., 2024; Leplat et al., 2025). This resistance occurs through various molecular mechanisms that reduce the effectiveness of biocides against the target (Buffet-Bataillon et al., 2012; Wiederhold, 2017; Kim et al., 2018; Toreno et al., 2024). Some traditional antiseptics widely used to protect cultural heritage objects from fungi have negative impacts on materials, the environment, and human health (Sterflinger, 2010; Mohapatra et al., 2022; Arnold et al., 2023; Leplat et al., 2025), which, during the last few years, has significantly narrowed the spectrum of practically used compounds (Sterflinger and Piñar, 2013; Romani et al., 2022). Traditional approaches to discovering antiseptics are based on random screening and subsequent chemical optimization; in many cases, this strategy is ineffective. Here, we used a rational approach for the design of a new type of antiseptic, which is based on the use of a system of chemical regulators of certain metabolic transformations of amino acids, and demonstrated the efficiency of this approach. Aminoalkyl *H*-phosphinic acids of the general formula (I) are the structural analogs of natural amino acids (II) and are used as a source of compounds with fungicidal activity (Scheme 1).

The single-charged *H*-phosphinic group of AA-P_H_ has a flattened tetrahedral geometry due to the smaller volume of the hydrogen atom, as suggested by crystallographic data for the distal *H*-phosphinic analog of aspartate (Asp-β-P_H_, Figure 2) (Schwalbe et al., 1993). The free rotation of the *H*-phosphinic group can ensure such an orientation of this hydrogen atom in the substrate-binding site, thereby providing a minimal steric effect. Therefore, the *H*-phosphinic group is a bioisostere of the flat single-charged carboxyl group, as confirmed by several substrate-like enzymatic transformations of α-amino-*H*-phosphinic acids. The enzymatic transformations and inhibitory activities of AA-P_H_ listed in Table 1 are worth adding to the kinetic resolution of *rac*-Glu-γ-P_H_ using *E. coli* glutamate decarboxylase, which yielded *D*-Glu-γ-P_H_ and *H*-phosphinic analogs of GABA. The latter is further transformed into the *H*-phosphinic analog of succinate by GABase, a commercial crude preparation of *Pseudomonas fluorescens* containing GABA transaminase and succinic semialdehyde dehydrogenase (De Biase et al., 2020). Therefore, the antifungal activity of AA-P_H_ compounds likely depends on their ability to interfere with key metabolic pathways in fungi; for example, Ala-P_H_ may be intracellularly transaminated to a phosphinic analog of pyruvate (Pyr-P_H_), which inhibits pyruvate dehydrogenase (Laber and Amrhein, 1987) and thereby reduces the levels of acetyl-CoA and malonyl-CoA, affecting pigment biosynthesis and energy metabolism.

The color change in the mycelium of *P. chrysogenum* STG-117 treated with Ala-P_H_, Asp-α-P_H_, or Val-P_H_ (Figure 5) is of interest. The analysis of possible metabolic transformations of AA-P_H_ (Figure 2) is complicated by insufficient knowledge about the metabolism of mold fungi. However, it is possible to suggest some explanations for the bleaching activity of the *H*-phosphinic analogs of these three amino acids.

**Figure 12:**
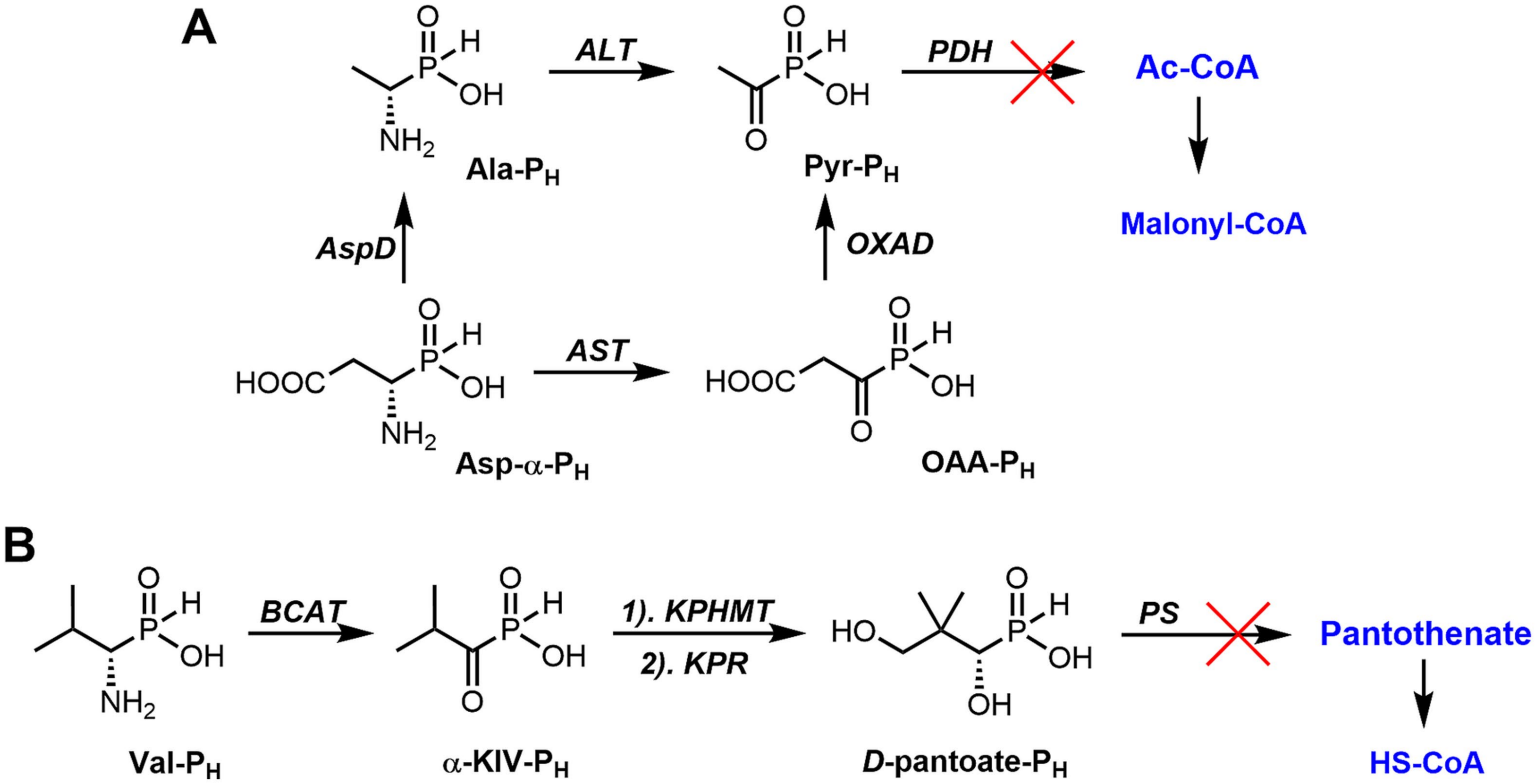
Putative mechanism of bleaching of *P. chrysogenum* colonies treated with some *H*-phosphinic analogs of natural amino acids. **(A)** Effect of Ala-P_H_ and Asp-α-P_H_ on Ac-CoA biosynthesis; **(B)** effect of Val-P_H_ on HS-CoA biosynthesis. AspD, Aspartate β-decarboxylase; ALT, Alanine aminotransferase; PDH, Pyruvate dehydrogenase; AST, Aspartate aminotransferase; OXAD, Oxaloacetate decarboxylase; BCAT, Branched-chain amino acid aminotransferase; KPHMT, Ketopantoate hydroxymethyltransferase; KPR, Ketopantoate reductase; PS, Pantothenate synthetase.

The condensations of acetyl-CoA (Ac-CoA) and malonyl-CoA, leading to the formation of aromatic systems and polyketides, are one of the key steps in the biosynthesis of the pigments sorbicillin and chrysogine in *P. chrysogenum* (Supplementary Figure S3). Therefore, the inhibition of the biosynthesis of Ac-CoA, malonyl-CoA, and coenzyme A (HS-CoA) should lead to bleaching of the mycelium of this mold fungus. Once in STG-117, Ala-P_H_ is known to be intracellularly transaminated into the *H*-phosphinic analog of pyruvate (Pyr-P_H_) (Laber and Amrhein, 1987), which is a very effective irreversible inhibitor of pyruvate dehydrogenase (Nemeria et al., 2006). Respectively, this will reduce the acetyl-CoA pool and lead to bleaching of the mycelium (Figure 12). This is in line with earlier data demonstrating that the treatment of the phytopathogenic fungus *Pyricularia oryzae* with Ala-P_H_ causes bleaching of the fungus mycelium (Zhukov et al., 2004b). Asp-α-P_H_ is enzymatically converted to oxaloacetate (Khurs et al., 1989), which, after subsequent decarboxylation, may yield Pyr-P_H_. An alternative pathway for the conversion of Asp-α-P_H_ to Pyr-P_H_ is also a two-step process. First, Asp-α-P_H_ may be converted to Ala-P_H_ by PLP-dependent aspartate β-decarboxylase (AspD, Figure 12), similar to Glu-γ-P_H_, which is decarboxylated by PLP-dependent glutamate decarboxylase (De Biase et al., 2020). The resulting Ala-P_H_ is known to be a substrate of PLP-dependent alanine aminotransferase (ALT, Figure 12), yielding Pyr-P_H_ (Laber and Amrhein, 1987). Treatment of STG-117 with Asp-α-P_H_ results in the biosynthesis of Pyr-P_H_, subsequent depletion of the Ac-CoA pool, and bleaching of the colonies (Figure 12). The currently suggested mechanism (Figure 12) explaining the bleaching effects of Ala-P_H_ and Asp-α-P_H_ is based mainly on their substrate-like transformations catalyzed by the corresponding enzymes from different sources.

Valine is a precursor of pantothenate and, respectively, HS-CoA (Leonardi and Jackowski, 2007). Despite the interaction of Val-P_H_ with branched-chain amino acids, transaminase has never been studied. The formation of the *H*-phosphinic analog of α-ketoisovalerate (α-KIV-P_H_, Figure 12) in STG-117 is expected, based on the known substrate-like transformations of different AA-P_H_, catalyzed by PLP-dependent transaminases (Laber and Amrhein, 1987; Khurs et al., 1989). Based on the substrate properties of the *H*-phosphinic analog of tyrosine in the PLP-dependent tyrosine-gamma-lyase reaction (Faleev et al., 2000) and Met-P_H_ in the PLP-dependent methionine-gamma-lyase reaction (Faleev et al., 2009), as well as on the substrate properties of SAM-P_H_ in the Dnmt3a reaction (Table 1) and in the catechol-*O*-methyl transferase reaction (Rudenko et al., 2024), it is highly possible that α-KIV-P_H_ may undergo transformation in the radical and be converted into *D*-pantoate-P_H_ (Figure 12). Thus formed, *D*-pantoate-P_H_ will not be the substrate of pantothenate synthetase, because the transition state of the *H*-phosphinic group (trigonal bipyramid) is noncomplementary to the active site of the enzyme, which is adapted to the tetrahedral intermediate of the carboxylic group. Since valine is an essential amino acid, in experiments on synthetic Czapek–Dox agar medium, the fungi will uptake Val-P_H_, which will result in the inhibition of pantothenate biosynthesis and subsequent bleaching of *P. chrysogenum* colonies (Figure 12).

The metabolism of methionine is not as variable as that of many other amino acids. In addition to its role in protein biosynthesis, this essential amino acid is the only precursor of *S*-аdenosyl-*L*-methionine (SAM), which is the second most abundant cofactor in living systems after ATP. SAM-mediated methylation controls the function of biomolecules and regulates numerous vital intracellular processes in all living organisms, including mold fungi. Met-P_H_, along with Ala-P_H_, was among the most effective inhibitors of the growth of the test STG strains in the experiments performed on Czapek–Dox agar medium (Figures 3, 4). Moreover, in the experiments on mock-up layers, Met-P_H_ completely inhibited the growth of all 11 test STG strains and was more active than traditional antiseptics, benzalkonium chloride (BAC), or sodium pentachlorophenolate (NaPCP), as depicted in Figure 11 and Supplementary Figure S2. This is in line with the excellent fungicidal activity of Met-P_H_ (equal to the Japanese fungicide, Fujione®) in field trials against *P. oryzae*, which is the cause of the most widespread rice blast disease (Zhukov et al., 2004a). The biological activity of Met-P_H_ is most likely related to its effects on biomethylation processes. Very recently, we demonstrated that the *H*-phosphinic analog of *S*-adenosylmethionine (SAM-P_H_) can be synthesized enzymatically from *L*-Met-P_H_ using *S*-adenosylmethionine synthetase (Rudenko et al., 2024). This is in line with earlier data on the biosynthesis of SAM-P_H_ in L1210 cells growing in the presence of non-toxic concentrations of *L*-Met-P_H_—this experiment was performed using a minimal growth-supporting concentration of natural methionine in the media (Khomutov et al., 2000). SAM-P_H_ is a functionally active mimetic of SAM, but it is slightly less effective than SAM in methyltransferase reactions catalyzed by catechol-*O*-methyltransferase (Rudenko et al., 2024) and DNA methyltransferase Dnmt3а, establishing methylation patterns in mammals (Filonov et al., 2023). SAM-P_H_ cannot effectively serve as a methyl group donor in all reactions catalyzed by methyltransferases. For example, SAM-P_H_ is not a substrate of the DNA-methyltransferase Dnmt1, which maintains DNA methylation during replication (Filonov et al., 2025). This would certainly be true for some other methylases, and the fungicidal activity of Met-P_H_ is most likely determined by its transformation into SAM-P_H_ and insufficient methylation of biomolecules. However, direct determination of SAM-P_H_ in the studied fungi is needed to confirm that Met-P_H_ is an original prodrug affecting methylation processes *via* the formation of SAM-P_H_.

The toxicity of AA-P_H_ remains unknown. The acute toxicity of Glu-γ-P_H_ toward mice and rats is in the range of several grams per kg of body weight (Takara et al., 1982). Some *H*-phosphinic analogs of amino acids have good antibacterial activity in minimal, but not rich, media, which can be exemplified by the activity of Glu-γ-P_H_ (De Biase et al., 2020). Compounds of this class do not exhibit activity against eukaryotic cells because they compete with amino acids present in the nutrient media. Low toxicity toward mammals is expected. However, long-term studies of material compatibility and detailed toxicological studies are still required before the systematic practical application of Met-P_H_ and related compounds in restorative dentistry.

## Conclusion

Our results clearly showed that at least some *H*-phosphinic analogs of natural amino acids are promising antiseptics for protecting paintings from biodeterioration. All 12 studied compounds inhibited the growth of fungi-destructors of painting material on agarized Czapek–Dox medium, although the activity varied. The four most promising analogs were added to sturgeon glue in the manufacture of layouts. Spectral analysis and analysis of the surface properties of the prepared layouts showed that the addition of Met-P_H_ did not cause significant changes compared to the controls. The addition of other *H*-phosphinic analogs of amino acids to the layouts induced small changes in the surface, according to AFM data. Met-P_H_ protected layouts from biodeterioration caused by fungi-destructors better than standard antiseptics currently used in paintings, such as benzalkonium chloride and sodium pentachlorophenolate. The range of antiseptics used to protect artworks has been significantly reduced over the years; therefore, it is necessary to search for new antiseptics that do not damage artworks and have high protective activity because fungi-destructors have developed resistance toward common fungicides. *H*-phosphinic analogs of natural amino acids may inhibit the growth of fungi *per se* and also undergo intracellular transformations into new phosphorus-containing derivatives, targeting different metabolic pathways. This allows these compounds to be considered promising instruments against resistant strains and makes the *H*-phosphinic analogs of natural amino acids a long-lasting tool to protect the artwork. In addition, these compounds are water-soluble, which is their essential benefit, as they can be used in the treatment of numerous hydrophilic painting materials.

## Data Availability

The original contributions presented in the study are included in the article/Supplementary material, further inquiries can be directed to the corresponding authors.

## Supplementary material

Supplementary Figure S1 AFM images of mock layers No. I–No. VII. Scanning area (from left to right) – 2 × 2, 10 × 10, and 20 × 20 μm. Sturgeon glue additives: **(A)** Gly-P_H_ (1-aminomethyl-*H*-phosphinic acid); **(B)** Met-P_H_ (racemic 1-amino-3-methylthiopropyl-*H*-phosphinic acid); – cocktail of Asp-α-P_H_ (racemic l-amino-2-carboxyethyl-*H*-phosphinic acid) + Asp-β-P_H_ (racemic 2-amino-2-carboxyethyl-*H*-phosphinic acid); **(D)** cocktail of Gly-P_H_ + Met-P_H_ + Asp-α-P_H_ + Asp-β-P_H_; **(E)** benzalkonium chloride (BAC); **(F)** sodium pentachlorophenolate (NaPCP); **(G)** without additives (control). N/D, no data (since it is impossible to carry out AFM with this material in such a scanning zone due to the large differential high).

Supplementary Figure S2 Growth of test cultures on mock-ups with the addition of selected antifungal compounds (from left to right): BAC, benzalkonium chloride; NaPCP, sodium pentachlorophenolate; Met-P_H_ (racemic 1-amino-3-methylthiopropyl-H-phosphinic acid). Forty days after inoculation, 26 °C. Strains: *Aspergillus versicolor* STG-25G, *Simplicillium lamellicola* STG-96, *A. creber* STG-57, *Cladosporium halotolerans* STG-52B, *A. versicolor* STG-86, *Microascus paisii* STG-103, *A. creber* STG-93W, *C. parahalotolerans* STG-93B, *A. protuberus* STG-106, and *Ulocladium* sp. AAZ-2020a STG-36.

Supplementary Figure S3 General pathways of sorbicillin **(A)** and chrysogine **(B)** biosynthesis.
