# Supplementary figures and images for: H-phosphinic analogs of natural amino acids: a novel and efficient treatment for preventing biodeterioration of treasured painted artworks
Source: Front Microbiol. 2026 Apr 2;17:1677277. doi: 10.3389/fmicb.2026.1677277 (PMC13085325; doi:10.3389/fmicb.2026.1677277)

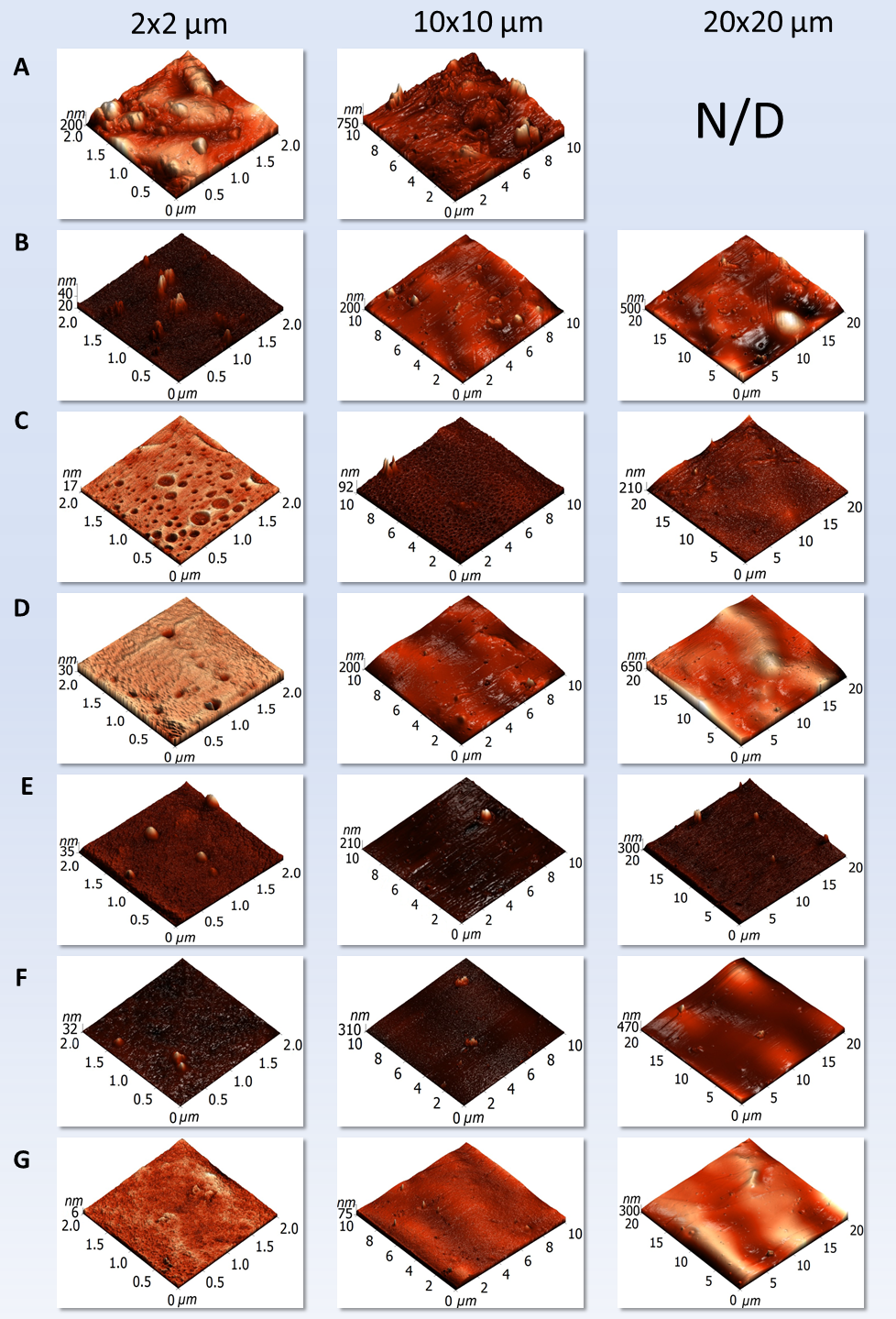

Supplement: Supplementary Figure S1 — AFM images of mock layers No. I–No. VII. Scanning area (from left to right) – 2 × 2, 10 × 10, and 20 × 20 μm. Sturgeon glue additives: (A) Gly-PH (1-aminomethyl-H-phosphinic acid); (B) Met-PH (racemic 1-amino-3-methylthiopropyl-H-phosphinic acid); – cocktail of Asp-α-PH (racemic l-amino-2-carboxyethyl-H-phosphinic acid) + Asp-β-PH (racemic 2-amino-2-carboxyethyl-H-phosphinic acid); (D) cocktail of Gly-PH + Met-PH + Asp-α-PH + Asp-β-PH; (E) benzalkonium chloride (BAC); (F) sodium pentachlorophenolate (NaPCP); (G) without additives (control). N/D, no data (since it is impossible to carry out AFM with this material in such a scanning zone due to the large differential high). [file Image_1.tif]

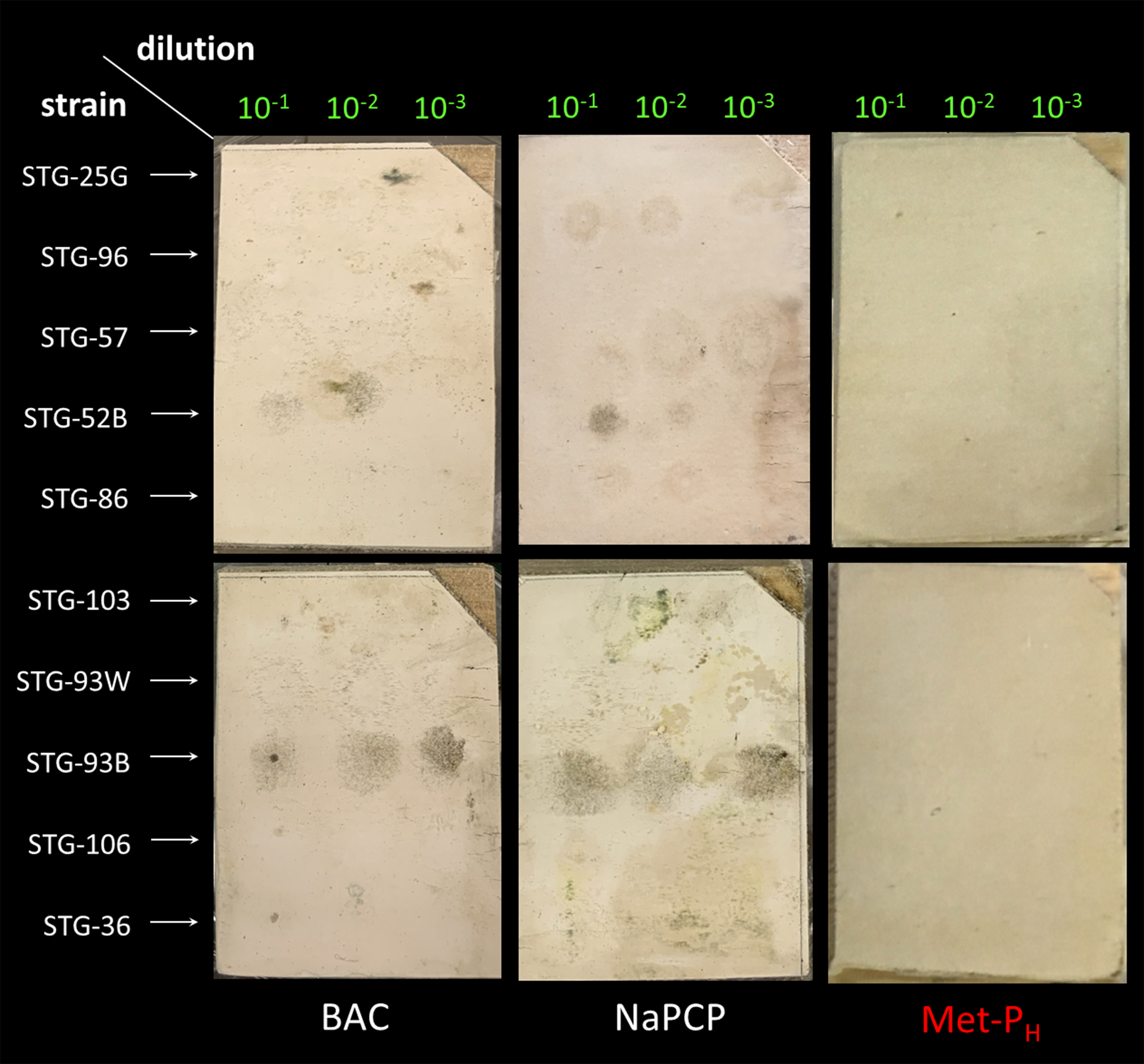

Supplement: Supplementary Figure S2 — Growth of test cultures on mock-ups with the addition of selected antifungal compounds (from left to right): BAC, benzalkonium chloride; NaPCP, sodium pentachlorophenolate; Met-PH (racemic 1-amino-3-methylthiopropyl-H-phosphinic acid). Forty days after inoculation, 26 °C. Strains: Aspergillus versicolor STG-25G, Simplicillium lamellicola STG-96, A. creber STG-57, Cladosporium halotolerans STG-52B, A. versicolor STG-86, Microascus paisii STG-103, A. creber STG-93W, C. parahalotolerans STG-93B, A. protuberus STG-106, and Ulocladium sp. AAZ-2020a STG-36. [file Image_2.tif]

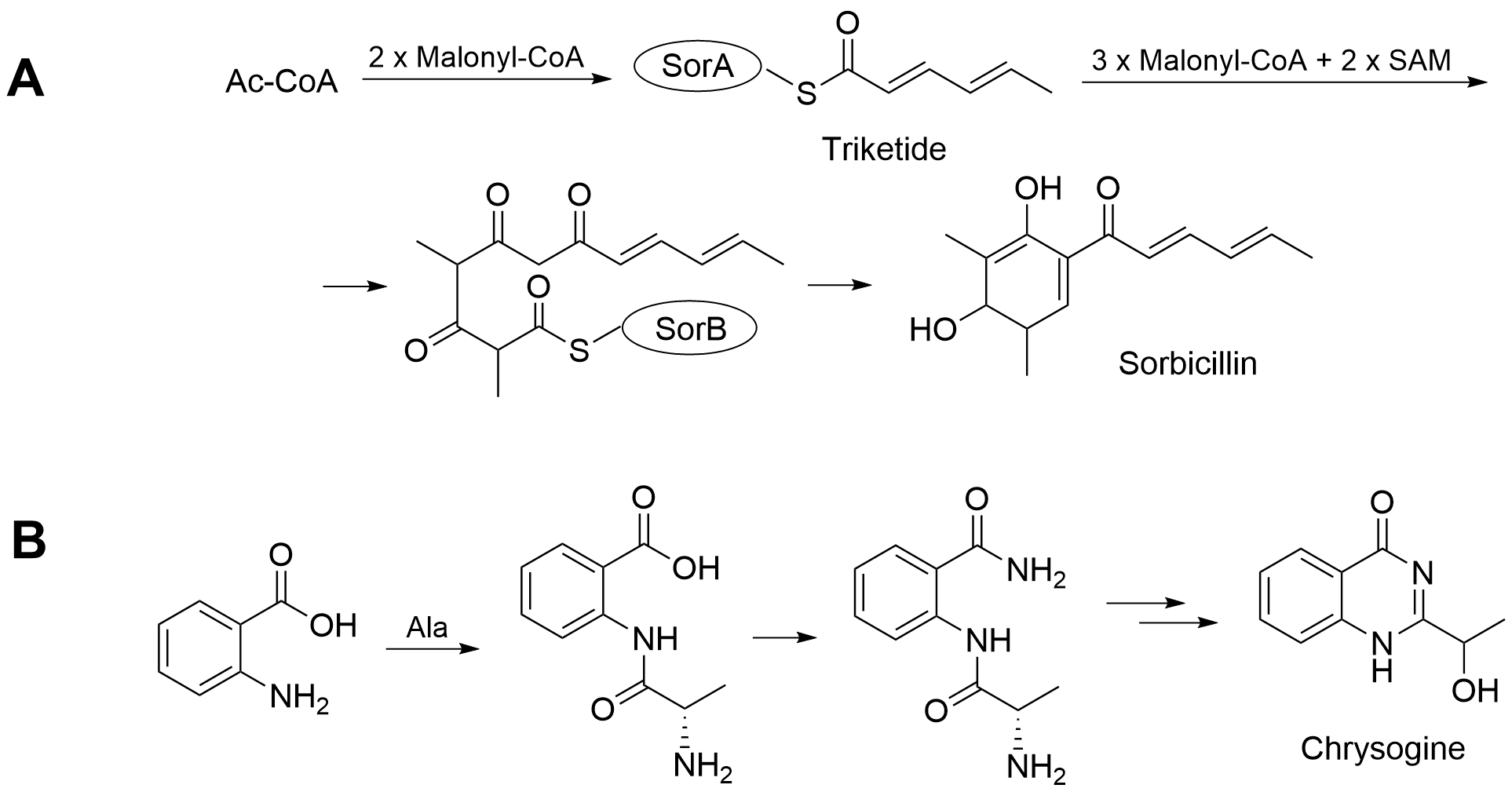

Supplement: Supplementary Figure S3 — General pathways of sorbicillin (A) and chrysogine (B) biosynthesis. [file Image_3.tif]
